# A novel Ru(II) complex with pyrene-1-carbaldehyde hydrazone: structural, electronic, theoretical, and biological studies

**DOI:** 10.3389/fchem.2026.1904958

**Published:** 2026-08-31

**Authors:** Stefan Perendija, Dušan Dimić, Dejan Milenković, Djura Nakarada, Dragoslava Djikić, Tijana Subotički, Silvia Chichiarelli, Goran N. Kaluđerović, Jasmina Dimitrić Marković

**Affiliations:** 1 Faculty of Physical Chemistry, University of Belgrade, Belgrade, Serbia; 2 Institute for Information Technologies, University of Kragujevac, Kragujevac, Serbia; 3 Institute for Medical Research, University of Belgrade, Belgrade, Serbia; 4 Department of Biochemical Sciences A. Rossi Fanelli, University of Rome La Sapienza, Rome, Italy; 5 Department of Engineering and Natural Sciences, University of Applied Sciences Merseburg, Merseburg, Germany

**Keywords:** anticancer activity, DFT, hydrazone ligand, molecular docking, molecular dynamics simulation, protein and DNA binding, QTAIM analysis, ruthenium(II) arene complex

## Abstract

A new Ru(II) half-sandwich complex, **1**, containing an η^6^-*p*-cymene ligand and (*E*)-1-(pyren-1-ylmethylidene)hydrazine, was synthesized in good yield and characterized by FTIR, NMR, elemental analysis, and density functional theory calculations. The close agreement between experimental and theoretical FTIR, ^1^H NMR, and ^13^C NMR data confirmed the proposed structure, while geometry optimization revealed the expected pseudo-octahedral piano-stool arrangement around the Ru(II) center. QTAIM analysis indicated that the metal–ligand interactions, particularly Ru–N and Ru–arene bonds, possess partial covalent character and play a key role in stabilizing the complex. Biological interaction studies showed that **1** binds spontaneously to human serum albumin (HSA) with moderate affinity via predominantly hydrophobic interactions and also binds calf thymus DNA, as demonstrated by spectrofluorimetric titration and ethidium bromide displacement experiments, suggesting mixed intercalative and groove-binding behavior. Molecular docking revealed that **1** binds most strongly to BCL-2/BCL-xL among all investigated targets, while also showing good affinity toward JAK2, KRAS, and BRAF. The docking protocol was supported by the close agreement between calculated and experimental binding free energies for HSA and DNA. Molecular dynamics and MM/PBSA analyses further confirmed that the **1**–BCL-2/BCL-xL complex is stable and thermodynamically favorable, indicating that this anti-apoptotic protein may represent the most relevant biological target of complex **1**. EPR measurements revealed selective radical-scavenging activity, with pronounced efficiency toward hydroxyl radicals but much weaker effects against DPPH, superoxide, and ascorbyl radicals. *In vitro* studies further demonstrated cytotoxic activity of the complex in several cancer cell lines, with the strongest effect observed in MIA PaCa-2 cells, accompanied by increased lipid peroxidation, protein oxidation, and alterations in matrix metalloproteinase activity, indicating that oxidative stress may contribute to its biological effects. The results identify **1** as a structurally well-defined Ru(II) compound with promising protein- and DNA-binding properties, selective antioxidant behavior, and moderate anticancer potential.

## 1 Introduction

Cancer remains one of the leading causes of mortality worldwide and continues to represent a major challenge for modern medicine. Conventional therapeutic strategies, including surgery, radiotherapy, and chemotherapy, constitute the primary approaches in clinical oncology and are often applied either individually or in combination depending on tumor type and stage. In recent years, substantial progress in biomedical research has led to the development of additional therapeutic strategies to improve treatment selectivity and efficacy. Among these approaches, immunotherapy has emerged as a powerful method that enhances the ability of the immune system to recognize and eliminate malignant cells (Ling et al., 2022; Kong and Chen, 2022; Pham et al., 2018; Baker et al., 2018).

Metal-based drugs play an important role in contemporary medicinal chemistry, particularly in anticancer research. Certain metal complexes function as theranostic agents, integrating diagnostic and therapeutic functions within a single molecular framework. Such systems can participate in imaging processes while simultaneously exerting therapeutic effects, enabling improved monitoring and treatment of malignant diseases. Among transition-metal compounds investigated for biomedical applications, ruthenium complexes have attracted considerable interest owing to their rich coordination chemistry and promising biological properties. Notably, numerous ruthenium-based compounds have demonstrated the ability to overcome resistance mechanisms associated with platinum-based chemotherapeutics, highlighting their potential as alternative or complementary anticancer agents (Kenny and Marmion, 2019; Thota et al., 2018; Bhattacharya et al., 2025; Zhang et al., 2022).

Although organometallic complexes are frequently considered sensitive to environmental factors such as air or moisture, a variety of ruthenium compounds have been developed that exhibit remarkable stability under physiological conditions. Many ruthenium-based complexes remain stable in aqueous and alcoholic media and display relatively low sensitivity toward oxygen and sulfur-containing biomolecules. These characteristics contribute to their suitability for applications in biological and medicinal systems (Crabtree, 2009).

In biological environments, ruthenium most commonly exists in the oxidation states Ru(II) and Ru(III). The reduced Ru(II) species generally exhibits higher kinetic reactivity and an enhanced ability to interact with biological targets. Both oxidation states typically adopt six-coordinate octahedral geometries, although they can accommodate a wide range of ligands and coordination modes. This structural flexibility, together with their accessible redox chemistry, enables ruthenium complexes to participate in various biologically relevant processes and contributes to their significant potential in medicinal applications (Jabłońska-Wawrzycka. et al., 2013; Strasser et al., 2015).

The ligand-substitution kinetics of Ru(II) complexes occur on timescales comparable to those observed for platinum (II) compounds, leading to consideration of ruthenium as a promising alternative to platinum-based chemotherapeutics. In addition, many ruthenium complexes display lower systemic toxicity while maintaining significant biological activity. Several compounds also exhibit some selectivity toward malignant cells. This behavior has been partly attributed to the ability of ruthenium to mimic iron in biological systems. Since rapidly proliferating tumor cells frequently overexpress transferrin receptors to meet their increased iron requirements, these pathways may facilitate the preferential uptake and accumulation of ruthenium complexes within tumor tissues (Reedijk, 2008; Gasser. and Metzler-Nolte, 2012; Brabec and Nováková, 2006; Lv et al., 2024; Gatter et al., 1983).

Among the different classes of ruthenium compounds investigated for anticancer applications, half-sandwich Ru(II)–arene complexes have attracted particular attention. Complexes incorporating the ligand 1,3,5-triaza-7-phosphatricyclo [3.3.1.1]decane (PTA), commonly referred to as the RAPTA compound, represent one of the most extensively studied families of such systems. Their biological properties can be readily tuned through rational modification of the ligand environment. Structurally, these complexes typically adopt the characteristic “piano-stool” geometry, in which an η^6^-coordinated arene ligand forms the “seat,” while a combination of mono- and bidentate ligands constitutes the three “legs” of the structure. The presence of chelating ligands often enhances the stability of the complex and can improve its interactions with biological targets. Furthermore, Ru(II)–arene complexes often exhibit a balance of hydrophilic and hydrophobic properties, which may facilitate favorable interactions in cellular environments. The combination of a stable Ru(II)–arene core and a versatile ligand framework enables the generation of a wide range of structurally diverse compounds and supports the rational design of new metal-based anticancer agents (Hongthong, et al., 2021; Pettinari et al., 2014; Süss-Fink, 2010; Swaminathan and Karvembu, 2023).

Ruthenium has therefore emerged as one of the most extensively investigated transition metals in the search for novel anticancer drugs. Several ruthenium-based complexes have progressed to clinical evaluation, including NAMI-A, KP1019, NKP1339, and the photosensitizer TLD-1433 (Alessio, 2017; Alessio and Messori, 2019; Bergamo, et al., 2009; Leijen et al., 2015). These compounds exhibit pharmacological properties and mechanisms of action that differ significantly from those of traditional platinum-based chemotherapeutics. For example, NAMI-A has been primarily recognized for its pronounced antimetastatic activity rather than strong cytotoxicity toward primary tumors, suggesting that ruthenium complexes may influence cancer progression through mechanisms distinct from direct DNA damage. In contrast, KP1019 and its sodium salt analog NKP1339 have demonstrated cytotoxic activity against a variety of tumor types and have been investigated in clinical studies for the treatment of advanced malignancies. More recently, TLD-1433 has been developed as a ruthenium-based photosensitizer for photodynamic therapy, further illustrating the versatility of ruthenium complexes in oncological applications (Lilge et al., 2020; Wang, et al., 2024). Collectively, these findings emphasize the considerable therapeutic potential of ruthenium compounds and their growing importance in the development of new metal-based anticancer agents.

In this study, the synthesis and comprehensive characterization of a new Ru(II) arene complex containing the hydrazone ligand (*E*)-1-(pyren-1-ylmethylidene) hydrazine are presented. The structural and electronic properties of the complex were investigated using spectroscopic techniques in combination with density functional theory calculations and QTAIM analysis. In addition, molecular docking and molecular dynamics simulations were performed to provide deeper insight into the interactions of the complex with biologically relevant macromolecular targets. The biological behavior of the complex was further examined through experimental studies of its binding to human serum albumin and DNA, evaluation of radical-scavenging activity by EPR spectroscopy, and assessment of cytotoxic effects in several cancer cell lines.

## 2 Materials and methods

### 2.1 Materials and instrumentation

The ligand, 1-pyrenecarboxaldehyde hydrazone (1-PCAH), was synthesized according to the procedure outlined by Liang and coworkers (Liang et al., 2024). Hydrazine hydrate, purchased from Sigma–Aldrich Co., LLC (St. Louis, MO, United States), and [{RuCl2(η^6^-*p*-cymene)}_2_], sourced from Tokyo Chemical Industry Co. (TCI, Tokyo, Japan), served as starting materials. Tetrahydrofuran (THF) and diethyl ether were purchased from Carl Roth. Solvents were dried over suitable molecular sieves for at least 2 weeks before use to remove residual moisture. The synthesis was performed using the Schlenk technique.

Elemental analysis was performed on a Thermo Scientific Flash Smart CHNS (Italy). Deuterated solvents, DCM-d_2_ and THF-d_4_, were purchased from Deuterosolvents GmbH and employed in NMR analysis. Proton ^1^H and ^13^C NMR spectra, recorded on a Bruker Avance™ 400 MHz spectrometer at 400.23 MHz and 100.1 MHz, respectively, were used to characterize the synthesized complex. FTIR spectrum (ATR technique) was recorded on a Bruker IFS 66v/s FTIR spectrometer.

### 2.2 Synthesis of dichlorido ((E)-1-(pyren-1-ylmethylidene)hydrazine-κN) (η6-p-cymene)ruthenium (II) complex (1)

In a 50 mL flask, [{RuCl_2_(η^6^-*p*-cymene)}_2_] (50 mg, 0.08 mmol) and 1-pyrenecarboxaldehyde hydrazone (49.9 mg, 0.20 mmol) were added and suspended in THF (25 mL) at room temperature. The mixture was thoroughly stirred and degassed by bubbling nitrogen through a syringe for 15 min. A yellow suspension was formed as a fine precipitate in the reaction. The reaction was allowed to proceed overnight, after which diethyl ether (Et_2_O) was added to the yellow suspension. The suspension was filtered to collect the solid, which was washed 3 times with diethyl ether (3 × 5 mL) and air-dried. The yield was 54.9 mg, corresponding to 62.3%. ^1^H and ^13^C NMR spectra are included in Supplementary Figures S1 and S2.

Anal. Calcd for C27H26Cl2N2Ru (550.49): C, 58.91; H, 4.76; N, 5.09. Found: C, 59.03; H, 4.71; N, 5.21.^1^H NMR (DCM-d_2_) δ 10.80 (s, CH, 1H), 9.48 (s, 1H), 8.75 (d, 3JH,H = 9.3 Hz, 1H), 8.59 (d, 3JH,H = 8.2 Hz, 1H), 8.37 – 8.19 (m, 6H), 8.17 – 8.04 (m, 3H), 6.43 (s, 2H), 5.50 (s, NH), 5.63 – 5.13 (d, 3JH,H = 6.2 Hz, CHCHcym, 4H), 3.00 (h, 3JH,H = 6.8 Hz, CH, 1H), 2.23 (s, CCH3, 3H), 1.24 (d, 3JH,H = 7.0 Hz, C(CH3)2, 6H). ^13^C NMR (CDCl_3_) δ 158.4 (HC = N), 132.2, 131.4, 130.7, 130.0, 129.3, 127.2, 127.1, 126.9, 126.5, 125.9, 125.3, 124.8, 123.0 (CAr, pyrene), 112.0, 99.1, 88.3, 81.3, 79.9, 73.0 (CAr, η^6^-*p*-cym), 31.3 (CH(CH3)2, *p*-cym), 22.8, 22.4, 17.6 (CH3, *p*-cym).

### 2.3 Theoretical calculations and QTAIM

The Gaussian 09 software package (Frisch et al., 2009), employing the B3LYP functional, 6‐311++G(d,p) basis set (used for H, C, N, and Cl atoms), and LanL2DZ basis set for Ru(II) ions (Becke, 1993; Dunning et al., 1989; Hay and Wadt, 1985), was used for structure optimization of **1**. Confirming the stability of the optimized geometry, the absence of imaginary frequencies in the Hessian matrix indicated that the structure corresponds to a local minimum on the potential energy surface.

For the prediction of NMR spectra and the calculation of ^1^H and ^13^C NMR chemical shifts, the structure of **1** was reoptimized in solvent using the Conductor-like polarizable Continuum Model (CPCM) (Marenich et al., 2009). The GIAO method (Bohmann et al., 1997) was used to determine chemical shielding, and chemical shifts were calculated relative to tetramethylsilane (TMS), which served as the reference compound.

The stabilization interactions within the complex structure were examined using the Natural Bond Orbital (NBO) and Quantum Theory of Atoms in Molecules (QTAIM) approaches. The NBO analysis (Foster and Weinhold, 1980) was performed in Gaussian 09. The QTAIM studies, as developed by Bader (Bader, 1985; Bader et al., 1983), were performed in the AIMAll program package (Keith, 2016), and they included the Bond Critical Points (BCPs) of the most prominent interactions. For this purpose, wfx files generated by Gaussian 09 were used.

### 2.4 Spectrofluorimetric analysis of complex-HSA binding affinity

The binding affinity of **1** toward HSA was investigated by spectrofluorimetric titration using a Cary Eclipse MY2048CH03 spectrophotometer (Agilent Technologies, Santa Clara, CA, United States). The excitation and emission slit widths were set to 10 nm and 5 nm, respectively, while the scan rate was maintained at 600 nm min^−1^. The experiments were carried out in phosphate-buffered saline (PBS) (pH 7.4), containing 137 mM NaCl and 2.7 mM KCl, with a final HSA concentration of 5 μM. Excitation of fluorophores was performed at 280 nm, selectively exciting the tryptophan residues within the HSA structure. Increasing concentrations of **1** (1–9 μM) were titrated into the protein solution, and the interaction was monitored by a progressive decrease in fluorescence intensity at 340 nm. Quantitative analysis of the quenching data was performed using the Stern–Volmer equation (Equation 1) and double-logarithm Stern–Volmer equation (Equation 2) to determine the binding affinity parameters.

$$\frac{F_{0}}{F}=1+K_{sv}\left[Q\right]$$ (1)

$$\log\left(\frac{F_{0}-F}{F}\right)=\log K_{b}+n\log\left[Q\right]$$ (2)

In the equation presented, F_0_ and F are the fluorescence emission intensities of the HSA solution without and with the added **1**, respectively; KSV is the Stern–Volmer quenching constant; K_b_ is the binding constant; and n is the number of binding positions. The changes in enthalpy and entropy of binding were calculated from the Van’t Hoff’s plot, which included binding constants determined at three temperatures (27, 32, and 37 °C) (Equation 3):

$$\ln K_{b}=-\frac{\Delta H_{b}}{R}+\frac{\Delta S_{b}}{R}$$ (3)

The change in Gibbs free energy of binding at three temperatures was calculated using the Gibbs-Helmholtz equation (Equation 4):

$$\Delta G_{b}=\Delta H_{b}-T\Delta S_{b}$$ (4)

### 2.5 Spectrofluorimetric determination of DNA binding affinity

The DNA-binding properties of **1** were investigated by fluorescence spectroscopy, monitoring the quenching of its intrinsic emission upon interaction with calf thymus DNA (CT-DNA). The concentration of CT-DNA was determined spectrophotometrically by measuring its absorbance at 260 nm, using a molar absorption coefficient of 6,600 dm^3^ mol^−1^ cm^−1^. All absorption measurements were performed on a Thermo Scientific Evolution 220 UV–Vis spectrophotometer (Thermo Fisher Scientific, Waltham, MA, United States).

For the fluorescence titration experiments, the concentration of **1** was kept constant at 1 × 10^−5−^ M. Fluorescence measurements were carried out with an excitation wavelength set at 294 nm, while the emission spectra were recorded in the range of 320–600 nm. Excitation and emission slit widths were fixed at 10 and 5 nm, respectively. A stock solution of CT-DNA (1.52 × 10^−4^ M) prepared in PBS was added stepwise to the solution of the **1**. After each addition of CT-DNA, the system was allowed to equilibrate for 2 min before recording the corresponding fluorescence spectrum.

In addition, spectrofluorimetric titration experiments were performed to evaluate the DNA-binding mode of **1** through ethidium bromide (EB) displacement studies. In these experiments, the concentrations of CT-DNA and EB were maintained constant at 50 μM and 5 μM, respectively, in phosphate-buffered saline at pH 7.4, containing 137 mM NaCl and 2.7 mM KCl. The concentration of **1** was gradually increased from 1 μM to 9 μM in 1 μM increments. Fluorescence measurements for the EB displacement assay were carried out using an excitation wavelength of 520 nm, and the emission spectra were recorded in the range of 540–650 nm. The excitation and emission slit widths were both set to 10 nm.

### 2.6 Molecular docking

#### 2.6.1 Receptor, DNA, and ligand preparation

Receptors and DNA structures were retrieved from the RCSB Protein Data Bank in PDB format (PDB IDs: 2BXG, 7JGW, 8BXH, 4QL3, 8C7X, 5HG8, 5OC7, and 152D) (Ghuman et al., 2005; Nguyen et al., 2021; Miao et al., 2024; Hunter et al., 2015; Arora et al., 2023; Cheng et al., 2016; Reckel et al., 2017; Lipscomb et al., 1994). Receptors were prepared by removing co-crystallized ligands, water molecules, and cofactors using BIOVIA Discovery Studio 4.0 (Dassault Systèmes 2021). Polar hydrogens were added, and Kollman partial charges were calculated with AutoDockTools (Morris et al., 2009). The prepared ligand, compound 1, was converted to the PDBQT format using OpenBabel (O’Boyle et al., 2011).

Docking grids were defined using AutoGridFR (Zhang et al., 2019) with the following grid centers (in Å) in x, y, z directions: HSA (8.380 × 2.245×-14.437), DNA (14.653 × 12.086 × 5.967), BCL-2/BCL-xL (−2.923×-2.139 × 22.232), JAK2 (−12.589 × 14.594 × 21.263) KRAS (83.179 × 31.230 × 14.136), BRAF (24.489 × 110.739 × 15.226), EGFR (12.724×-3.499×-29.992), and BCR-ABL (4.369×-49.248 × 92.430). Docking was performed with AutoDock-GPU (Santos-Martins et al., 2021) in rigid mode (200 GA runs, --nrun 200; --nev 2,500,000; --ngen 42,000; ADADELTA local search; population size 150). Binding energies were calculated in kcal/mol with AutoDock-GPU and converted to kJ/mol (multiplying by 4.184) for consistency with experimental data. The docking protocol and parameter choices followed the methodology described by Eichhorn et al. (2024) and Eichhorn et al. (2024) and the AutoDock suite (Morris et al., 2009).

#### 2.6.2 Molecular dynamics (MD) simulations

Docked **1** with BCL-2/BCL-xL served as the starting model for 100-ns MD simulations in explicit solvent. Solvation employed the TIP3P water model, and system neutrality was achieved by adding NaCl to a final concentration of 0.15 M. Energy minimization was performed with the steepest descent and conjugate gradient algorithms until convergence (tolerance = 1,000 kJ mol^−1^ nm^−1^). The system was equilibrated at physiological temperature (310.15 K) using a Berendsen weak coupling thermostat. Equilibration was conducted in the NVT ensemble for 2 ns, followed by a production run in the NPT ensemble for 100 ns? Constraints were applied via the LINCS algorithm, and temperature/pressure were controlled with a velocity-rescaling thermostat and Parrinello–Rahman barostat, respectively (Hess et al., 1997). All simulations were performed with GROMACS (version 2025.4) (Abraham et al., 2015), and trajectories were prepared for subsequent analysis.

#### 2.6.3 Ligand parameterization and ADME predictions

The **1** was parameterized with MCPB. py in the AmberTools suite (Li and Merz 2016), and the resulting parameters were converted to the GROMACS format for MD simulations. MM-PBSA binding energies were estimated with gmx_MMPBSA using snapshots from the MD trajectory to quantify energetics and validate docking results (Valdés-Tresanco et al., 2021; Miller et al., 2012). ADME descriptors were computed with a custom Python workflow based on RDKit, estimating properties such as LogP, molecular weight, hydrogen-bond donors/acceptors, topological polar surface area (TPSA), rotatable bonds, Lipinski violations, BBB permeation, and solubility, with toxicity predictions incorporated where applicable. The workflow aligns with established pipelines reported in recent literature (Simović et al., 2025).

### 2.7 EPR measurements

#### 2.7.1 DPPH radical scavenging assay

The antiradical capacity of **1** toward DPPH radicals was evaluated by electron paramagnetic resonance (EPR) spectroscopy (Jelisić et al., 2024). Briefly, 1 µL of a 10 µM sample solution (prepared in a water/DMSO mixture; final concentration 0.3 µM) was added to 29 µL of freshly prepared DPPH solution (210 µM in deionized water). After mixing, the reaction continued for 2 min at room temperature, after which the EPR spectrum was recorded. The control samples were prepared under the same conditions, except that the sample solution was replaced with the same volume of deionized water. All of the EPR spectra were acquired using a Bruker ELEXSYS-II E540 EPR spectrometer (Bruker, Rheinstetten, Germany) in X-band. The instrumental parameters were as follows: microwave frequency, 9.85 GHz; microwave power, 10 mW; modulation frequency, 100 kHz; and modulation amplitude, 0.1 mT.

Antioxidant activity (AA, %) was calculated according to Equation 5:

$$AA\left(\%\right)=100\times \frac{I_{c}-I_{a}}{I_{c}}$$ (5)

where I_c_ and I_a_ represent the double integer value of the two most prominent lines in the control and sample-containing spectra, respectively.

#### 2.7.2 Hydroxyl radical (HO^•^) scavenging assay

Hydroxyl radical scavenging was assessed using a Fenton reaction system in the presence of the spin trap DEPMPO (5-diethoxyphosphoryl-5-methyl-1-pyrroline-*N*-oxide) (Jelisić et al., 2024; Glavinić et al., 2025; Milosavljević et al., 2025). The reaction mixture (total volume 29 µL) consisted of 2 µL H_2_O_2_ (final concentration 0.35 mM), 1 µL DEPMPO (final concentration 3.5 mM), and 1 µL of the sample solution (10 μM; final concentration 0.3 µM), with deionized water added to volume. The reaction was initiated by adding 1 µL FeSO_4_ (final concentration 0.15 mM). The mixture was transferred into a gas-permeable Teflon tube and placed in the resonator cavity. The EPR spectrum of the DEPMPO/OH spin adduct was recorded after 2 min, under identical instrumental settings to those used for the DPPH measurements. The control experiments were performed by replacing the sample with the same volume of solvent.

#### 2.7.3 Superoxide anion (O_2_^•−^) scavenging assay

The scavenging activity toward superoxide radicals was evaluated using a DEPMPO-based spin trapping methodology (Glavinić et al., 2025). The reaction mixture contained 18 µL deionized water, 10 µL H_2_O_2_ (10 M), and 1 µL DEPMPO (100 mM). A 1 µL of the sample solution (10 μM; final concentration 0.3 µM) was added into this mixture. Radical formation was initiated by UV irradiation for 30 s, followed by 2 min incubation at room temperature. After that, the solution was placed into a gas-permeable Teflon tube for EPR analysis. A control sample was prepared in the same way, except that the sample solution was replaced with solvent. Spectra were recorded under the identical instrumental settings as for the DPPH measurements.

#### 2.7.4 Ascorbyl radical (^•^Asc) scavenging assay

The activity of the complex toward ascorbyl radicals was measured by monitoring the EPR signal intensity of ^•^Asc generated in DMSO (Milosavljević et al., 2025). A 10 µL of EDTA (final concentration 250 µM) and 1 µL FeCl_3_ (final concentration 8 µM) were mixed with 74 µL DMSO. After that, 5 µL of the sample solution (final concentration 0.5 µM) was added. The ascorbyl radical was generated by the addition of 10 µL ascorbic acid (final concentration 250 µM). After 2 min, 30 µL of the reaction mixture was transferred into a gas-permeable Teflon tube, and the EPR spectrum was recorded under the following parameters: microwave power 10 mW, microwave frequency 9.85 GHz, modulation frequency 100 kHz, and modulation amplitude 0.2 mT. The control measurements were conducted using the same volume of solvent instead of the sample. Antiradical activity was calculated as described for the DPPH assay.

### 2.8 UV–vis solution stability study

The stability of the **1** was evaluated in PBS/10% DMSO solutions using time-dependent UV-Vis spectroscopy on Evolution 220, Thermo Scientific UV-Vis Spectrophotometer over a period of 72 h. The **1** was initially dissolved in DMSO to prepare a stock solution (1 × 10^−3^ M). The stock solution was subsequently diluted with PBS buffer to obtain the working solution containing 10% DMSO and the 1 × 10^−5^ M concentration of the **1**. The electronic absorption spectra were recorded at predetermined time intervals (0, 30 min, 1, 2, 4, 6, 24, 48, and 72 h), using the corresponding PBS/10% DMSO solvent mixture as a blank.

### 2.9. Cell culture and biochemical assays

#### 2.9.1 Reagents and chemicals

Dulbecco’s Modified Eagle’s Medium (DMEM), penicillin-streptomycin solution, 0.25% trypsin/EDTA, and phosphate-buffered saline were purchased from Gibco. Fetal bovine serum (FBS) was purchased from Capricorn Scientific. MTT (3-(4,5-Dimethylthiazol-2-yl)-2,5-Diphenyltetrazolium Bromide) was purchased from Invitrogen, Thermo Fisher Scientific. Thiobarbituric acid and 1,1,3,3-tetramethoxypropane were purchased from Merck.

#### 2.9.2 Cell culture and experimental designs

The human breast cancer cell line (MDA-MB-231), human pancreatic cancer cell line (MIA PaCa-2), malignant melanoma (A-375), hepatocellular carcinoma cell line (HCC), erythroleukemia cell line (HEL 92.1.7.), and chronic myelogenous leukemia cell line (K-562) were obtained from the American Type Culture Collection (ATCC). The cells were cultured in 25 cm2 flasks with DMEM supplemented with 100 U/mL penicillin-streptomycin and 10% FBS at 37 °C with 5% CO_2_ in a humidified incubator. The complex was dissolved in DMSO to obtain a high-concentration solution (15 mM). Immediately before treatment, the solution was diluted in DMEM to obtain a working solution. In all cell viability experiments, the final DMSO concentration was maintained below 1.5%.

#### 2.9.3 MTT test

Cells were harvested when they reached 85%–90% confluent. 100 μL of cell suspension (15.000 cells per well) was added to the wells of the 96-well plate and incubated overnight to allow the cells to attach. The cells were treated with different concentrations of **1**, ranging from 20 to 600 µM. Untreated cells served as the control. After 24 h, 10 µL MTT (5 mg/mL) was added to each well. Cultures were incubated for 24 h at 37 °C. The reduction process was stopped by adding 100 µL/well of 10% SDS, acidified with 1 M HCl. Dissolution of the resulting blue color was continued overnight in the incubator at 37 °C. All treatments were performed in triplicate. The absorbance was read at 540 nm on an ELISA reader (RT-6100, Rayto, Shenzhen, China). The absorbance ratio of treated to untreated living cells was used to measure viability. Cytotoxic activity was measured as the IC_50_, the concentration required to reduce the absorbance of treated cells by 50% compared to the control (Mosmann 1983).

#### 2.9.4 Determination of the malondialdehyde and protein carbonyl concentrations

Each of the cell lines was treated with **1** at a concentration corresponding to the IC_50_ concentrations: A-375–233.6 µM, HCC – 94.2 µM for 24 h. Untreated cells were used as a control. The supernatants were collected for further analysis. Lipid peroxidation was estimated by a spectrophotometric assay based on the absorption maximum of the MDA complex with thiobarbituric acid in an acidic environment and high temperature. 1,1,3,3-Tetramethoxypropane was used as a standard. Absorbance was measured on the ELISA reader at 540 nm. The concentration was determined using a standard curve (Baskic et al., 2005). Determination of protein carbonyl (PC) concentration was based on the method of Levin and associates (Levine et al., 1990). Absorbance was read on the ELISA reader using a 375 nm filter.

#### 2.9.5 Detection of matrix metalloproteinase 2 and 9 activity

The cells were treated with **1** for 24 h at IC_50_ concentrations: A-375–233.6 µM, and HCC – 94.2 µM. Untreated cells were used as a control. The supernatants were collected for further analysis. Electrophoresis in a polyacrylamide gel containing 0.1% SDS and 0.2% gelatin was used to measure enzymatic activity. The gels were imaged with a ChemiDoc Imaging System (Bio-Rad Laboratories, Hercules, California) and estimated by densitometric scanning using the Image Lab (Bio-Rad Laboratories, Inc., Version 6.0.0.25) software tool.

#### 2.9.6 Statistical analysis

The One-way ANOVA and LSD *post hoc* test were applied using SPSS software for Windows (SPSS 16.0 for Windows Evaluation Version, SPSS Inc., United States). The results are expressed as the mean ± SEM, and differences with p < 0.05 are considered significant.

## 3 Results and discussion

### 3.1 Synthesis and spectral characterization of 1

**SCHEME 1:**
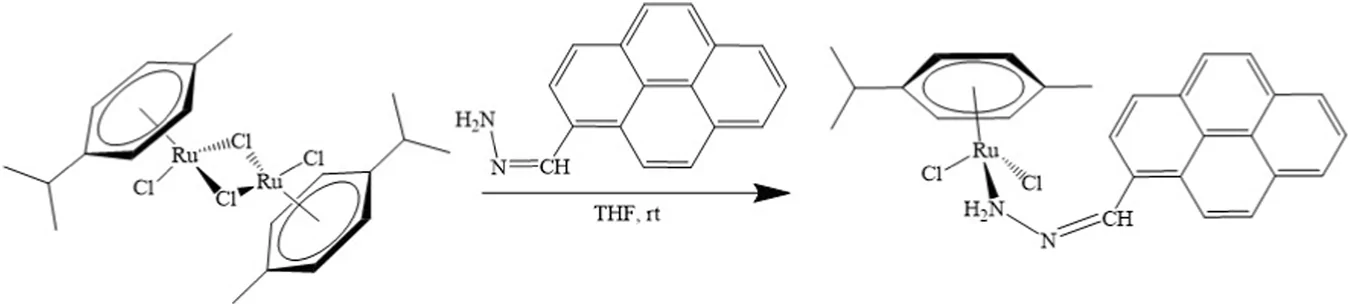
Synthesis of [RuCl_2_(η^6^-*p*-cymene) (1-pcah-κ*N*)].

Complex **1** was obtained from the reaction of the dimeric precursor [{RuCl_2_(η^6^-*p*-cymene)}_2_] with (*E*)-1-(pyren-1-ylmethylidene)hydrazine in tetrahydrofuran under an inert nitrogen atmosphere (Scheme 1). The reaction proceeded readily at room temperature, yielding a yellow product isolated in 62.3% yield. The formation of **1** was supported by spectroscopic FTIR and NMR analyses. The detailed assignment and discussion of the spectroscopic data are presented in the following subsections. In addition, elemental analysis confirmed the purity of **1**.

**FIGURE 1:**
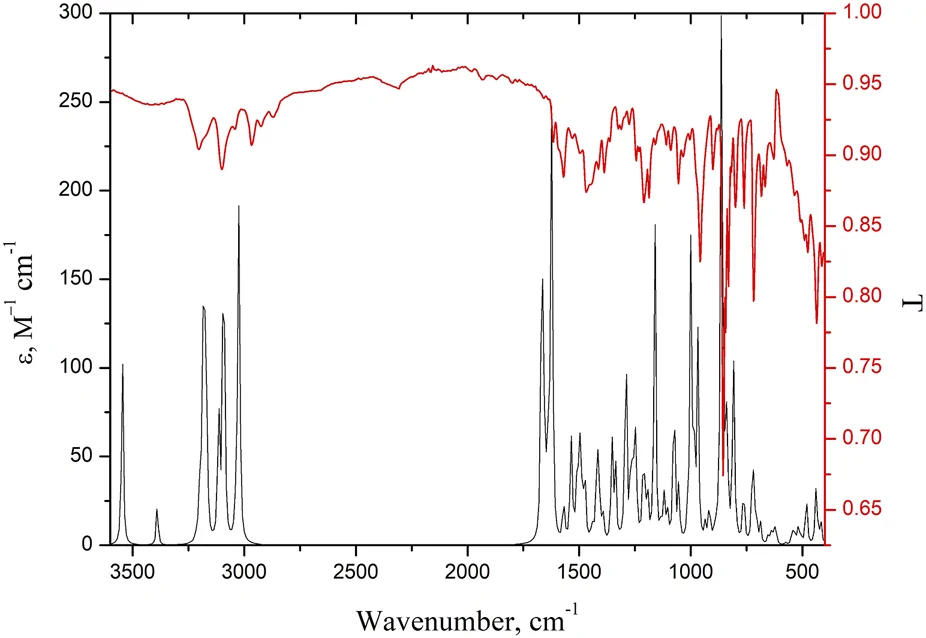
Experimental (red line) and theoretical (black line) FTIR spectra of [RuCl_2_(η^6^-*p*-cymene) (1-pcah-κ*N*)].

The experimental FTIR spectrum of **1** (Figure 1) exhibits the characteristic vibrational features expected for the proposed Ru(II) complex. Bands in the 3,172–3,186 cm^−1^ region are assigned to aromatic C–H stretching vibrations of the pyrene moiety, while a weak band at 3,210 cm^−1^ originates from aromatic C–H stretching contributions of the *p*-cymene ligand and the hydrazone fragment. A broad absorption between 3,097 and 3,000 cm^−1^ is attributed to aliphatic C–H stretching vibrations.

The most diagnostically relevant bands are observed below 1,670 cm^−1^. The bending vibration of the–NH_2_ group appears at 1,623 cm^−1^, whereas the C=N stretching vibration is observed at 1,584 cm^−1^, consistent with coordination of the hydrazone ligand to the Ru(II) center. Additional bands between 1,661 and 1,574 cm^−1^ arise from coupled C=C and C=N stretching vibrations of the aromatic and hydrazone frameworks. The intense band at 1,435 cm^−1^ is assigned to C=C stretching, while bands in the 1,512–1,489 cm^−1^ region correspond to C–H bending vibrations.

The amino-group in-plane bending vibration is observed at 1,266 cm^−1^, in agreement with literature data (Eichhorn et al., 2024), whereas the rocking mode appears at 896 cm^−1^. The shift of the amino-group vibrations toward lower wavenumbers is consistent with coordination of the nitrogen atom to the Ru(II) center. The N–N stretching vibrations are located at 1,010 cm^−1^, and the N–H twisting mode gives rise to an intense band at 727 cm^−1^. As expected for Ru(II) complexes, the Ru–N stretching vibration is observed below 560 cm^−1^ and is only weakly resolved in the spectrum.

The ^1^H NMR spectrum of **1** further supports the proposed structure (Supplementary Figure S1; Supplementary Table S1). The methyl protons of the isopropyl group resonate at 1.24 ppm. The methyl group of the *p*-cymene ligand appears at 2.23 ppm, while the corresponding methine proton is observed as a characteristic septet at 3.00 ppm. Aromatic protons of the *p*-cymene ligand give rise to a characteristic pattern between 5.13 and 5.63 ppm. The–NH_2_ hydrogen atoms are detected at 5.50 ppm. Resonances corresponding to the aromatic protons of the (*E*)-1-(pyren-1-ylmethylidene)hydrazine ligand are distributed over the downfield region between 8.28 and 10.80 ppm, reflecting their involvement in an extended conjugated system.

The ^13^C NMR spectrum of the Ru(II) complex displays a characteristic resonance at δ 158.44 ppm, assigned to the azomethine carbon (C=N) of the coordinated hydrazone ligand (Supplementary Figure S2; Supplementary Table S1). The relatively downfield position of this signal reflects the altered electronic environment of the imine group upon coordination to the ruthenium center. Signals corresponding to the pyrene aromatic carbons are observed in the region from 123.0 to 132.2 ppm, which is typical for a fused polyaromatic system and confirms the preservation of the pyrene framework within the complex. In addition, several resonances characteristic of the coordinated η^6^-*p*-cym ligand are present. Chemical shifts found between 73.0 and 112.0 ppm are assigned to the aromatic carbon atoms of the η^6^-*p*-cym ring. Furthermore, the resonances at 31.3, 22.8, 22.4, and 17.6 ppm correspond to the aliphatic carbons of the isopropyl and methyl substituents of the *p*-cym ligand.

### 3.2 Theoretical structural characterization of 1

Since repeated attempts to obtain single crystals of **1** for X-ray diffraction were unsuccessful, computational analysis was employed to provide insight into its molecular structure. The initial structural model was constructed based on closely related Ru(II) complexes reported in the literature (Dimitrić Marković et al., 2025; Eichhorn et al., 2023; Eichhorn et al., 2024), which exhibit similar coordination environments and structural motifs. Using these structural analogies as a guide, a reasonable starting geometry was generated and subsequently refined through DFT optimization.

**FIGURE 2:**
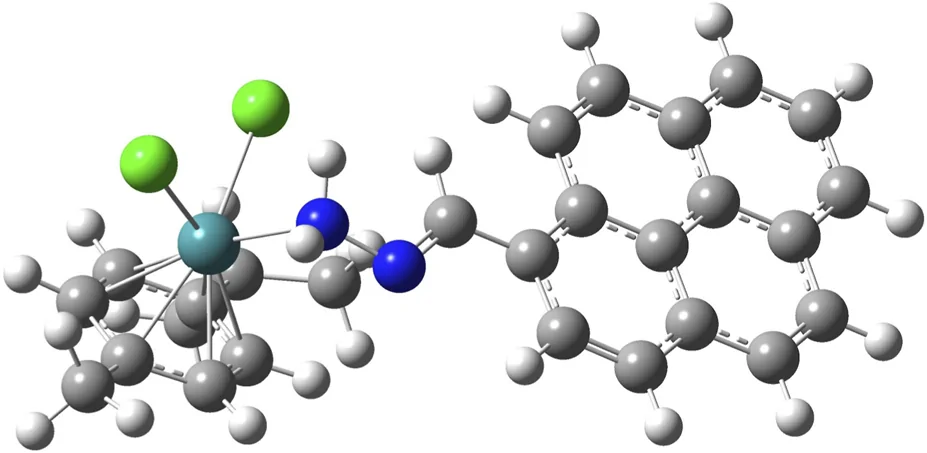
Optimized structure of [RuCl2(η6-p-cymene) (1-pcah-κN)] at the B3LYP/6-311++G(d,p) (H,C,N,Cl)/LanL2DZ (Ru) level of theory.

The geometry optimization was performed using the B3LYP functional with the 6-311++G(d,p) basis set for H, C, N, and Cl atoms, while the LanL2DZ basis set was applied for the Ru center (Eichhorn et al., 2023; Eichhorn et al., 2024). This computational approach has previously been shown to provide reliable structural parameters for related ruthenium complexes, yielding results that closely reproduce available crystallographic data (Dimitrić Marković et al., 2025). The optimized geometry of **1**, shown in Figure 2, provides a structural model consistent with the expected coordination features of this class of compounds.

The optimized structure of **1** reveals a coordination environment consisting of two chlorido ligands, an η^6^-bound *p*-cymene fragment, and an (*E*)-1-(pyren-1-ylmethylidene)hydrazine ligand. The calculated Ru–Cl bond lengths of 2.462 and 2.444 Å are in good agreement with values previously reported for structures of Ru(II) complexes containing *m*-nitrophenylhydrazine and *m*-chlorophenylhydrazine ligands (Eichhorn et al., 2024). In addition, crystallographic investigations of structurally related complexes have shown Ru–Cl distances in the range of 2.4007(15)–2.4481(14) Å (Dimitrić Marković et al., 2025; Eichhorn et al., 2023), confirming that the values obtained for **1** fall well within the expected range for this class of compounds.

The calculated Ru–N bond length of 2.172 Å is likewise consistent with those reported for comparable Ru(II) hydrazine complexes, whether optimized or crystallographically characterized (Eichhorn et al., 2024; Perendija et al., 2025). This observation suggests that variations in the substituent on the hydrazine ligand exert only a minor influence on the Ru–N bond distance. Such behavior can be rationalized by the presence of the N–N bond within the hydrazine framework, which limits extensive electronic delocalization across the ligand and therefore reduces the substituent effect on the metal–nitrogen interaction.

Structurally, the complex adopts the characteristic pseudo-octahedral “piano-stool” geometry typical of half-sandwich Ru(II)–arene complexes. In this arrangement, the η^6^-coordinated *p*-cymene ligand interacts with the Ru(II) center through three metal–carbon interactions. The average Ru–C bond distance was calculated to be 2.246 Å, which is comparable to values reported for related Ru(II)–arene complexes, including those containing hexamethylbenzene ligands (Lalrempuia et al., 2003). As is commonly observed in theoretical calculations, the optimized bond lengths are slightly longer than the crystallographic values, which is generally attributed to structural relaxation during geometry optimization of an isolated molecule in the gas phase.

The calculated Cl–Ru–Cl bond angle in **1** is 88.94°, closely matching those reported for analogous optimized structures containing *m*-nitrophenylhydrazine and *m*-chlorophenylhydrazine ligands, which were 90.09° and 90.32°, respectively (Dasmahapatra et al., 2024). A similar value (88.92°) has also been reported for a complex bearing a 3,4-dimethylphenylhydrazine ligand (Dimitrić Marković et al., 2025). In contrast, the calculated N–Ru–Cl bond angles of 79.88° and 82.34° show a more pronounced deviation from the ideal octahedral geometry. This distortion most likely arises from steric interactions between the coordinated ligands, particularly due to the bulky (*E*)-1-(pyren-1-ylmethylidene)hydrazine fragment, which imposes additional spatial constraints around the ruthenium center and slightly perturbs the coordination environment.

The theoretical IR spectrum of **1**, calculated without scaling factors, is shown in Figure 1 alongside the experimental spectrum, allowing direct comparison of band positions and relative intensities.

The calculated FTIR spectrum reproduces the main features of the experimental spectrum and supports the proposed structure of **1**. In the high-wavenumber region (>3,000 cm^−1^), the asymmetric and symmetric N–H stretching vibrations of the hydrazine unit are predicted at 3,546 and 3,390 cm^−1^, respectively, while the aromatic C–H stretching modes of the pyrene framework are reproduced within the 3,158–3,185 cm^−1^ range (exp. 3,172–3,186 cm^−1^). The calculated band at 3,184 cm^−1^ arises from overlapping contributions of the *p*-cymene and hydrazine fragments, whereas the methyl groups of the *p*-cymene ligand give rise to characteristic stretching vibrations at 3,094 and 3,021 cm^−1^.

In the mid-wavenumber region, the calculated–NH_2_ bending vibration (1,625 cm^−1^) is in excellent agreement with the experimental value (1,623 cm^−1^). The C=N stretching mode is predicted at 1,669 cm^−1^ (exp. 1,584 cm^−1^) and appears as a Fermi doublet, while the C=C stretching vibration is reproduced at 1,435 cm^−1^. The amino-group in-plane bending (1,291 cm^−1^), N–N stretching (1,010 cm^−1^; exp. 1,001 cm^−1^), and the twisting/rocking modes of the–NH_2_ group (709 cm^−1^; exp. 727 and 896 cm^−1^) are also satisfactorily reproduced, in agreement with previously reported data (Gulaczyk et al., 2003). The systematic red shift of the amino-group vibrations is consistent with coordination of the ligand to the Ru(II) center.

The low-frequency region further supports the proposed coordination environment. The Ru–N stretching vibration is calculated at 480 cm^−1^ (exp. <560 cm^−1^), while the Ru–Cl stretching modes appear at 278 and 269 cm^−1^, in good agreement with reported values for chlorido Ru(II) complexes (Eichhorn et al., 2023). These low-frequency modes are known to be sensitive to the coordination geometry and the nature of the chlorido ligands (Arlt et al., 2021), providing additional support for the proposed structure.

For comparison, theoretical ^1^H NMR chemical shifts were calculated for the DFT-optimized structure of **1**, including solvent effects through a polarizable continuum model for dichloromethane (Supplementary Table S1). The experimental and calculated values are summarized in Supplementary Table S1 and Supplementary Figure S1. The agreement between theory and experiment was quantified using the correlation coefficient (R) and the mean absolute error (MAE), which represents the average absolute deviation between calculated and experimental chemical shifts. A slight systematic overestimation of the calculated values is observed, consistent with the absence of explicit solute–solvent interactions in the computational model.

The methyl protons of the isopropyl group appear at 1.26 ppm (exp. 1.24 ppm), while the methyl group of the *p*-cymene ligand resonates at 2.65 ppm (exp. 2.23 ppm). The methine proton of the isopropyl group is observed as a septet at 3.03 ppm (exp. 3.00 ppm). In the aromatic region, the *p*-cymene protons form a multiplet centered at 5.13 ppm (exp. 5.38 ppm), and the–NH_2_ protons are assigned to the signal at 5.41 ppm (exp. 5.50 ppm). The aromatic protons of the (*E*)-1-(pyren-1-ylmethylidene)hydrazine ligand are distributed over the range 8.49–9.79 ppm (exp. 8.28–10.80 ppm).

The experimental and calculated values of ^13^C NMR resonance are summarized in Supplementary Table S1. The calculated ^13^C NMR spectrum of the complex shows resonances at 18.1, 18.8, and 21.8 ppm (exp. 17.6, 22.4, and 22.8 ppm), assigned to the methyl carbons of the isopropyl substituent and the methyl group in the para position. The methyne carbon atom of the isopropyl moiety was predicted at 35.7 ppm (exp. 31.3 ppm). The aromatic carbon atoms of the coordinated *p*-cymene ring exhibit calculated resonances at 83.9, 88.2, 93.5, 99.3, 104.8, and 116.3 ppm (exp. 73.0, 79.9, 81.3, 88.3, 99.1, and 112.0 ppm). Carbon atoms belonging to the 1-pyrenecarbaldehyde hydrazone ligand are calculated to resonate in the range 124.9–140.1 ppm (exp. 126.12–145.37 ppm), while the carbon atom directly bonded to the hydrazone nitrogen atom appears at the highest chemical shift value of 159.5 ppm (exp. 158.4 ppm) due to the strong deshielding effect of the electronegative nitrogen atom.

The comparison between the calculated and experimental ^13^C NMR chemical shifts reveals a satisfactory agreement, confirming the proposed structure of the complex and supporting the assignment of the carbon resonances. The observed deviations are generally small and can be attributed to solvent effects, intermolecular interactions, conformational flexibility in solution, and the inherent limitations of the computational model employed for the calculations.

Although single-crystal X-ray diffraction data could not be obtained despite repeated crystallization attempts, the excellent agreement between elemental analysis, FTIR, experimental and DFT-calculated NMR spectra, together with the optimized geometry and QTAIM analysis, provides strong evidence for the proposed molecular structure. However, crystallographic confirmation would provide additional structural validation and remains desirable in future studies.

### 3.3 QTAIM analysis

**FIGURE 3:**
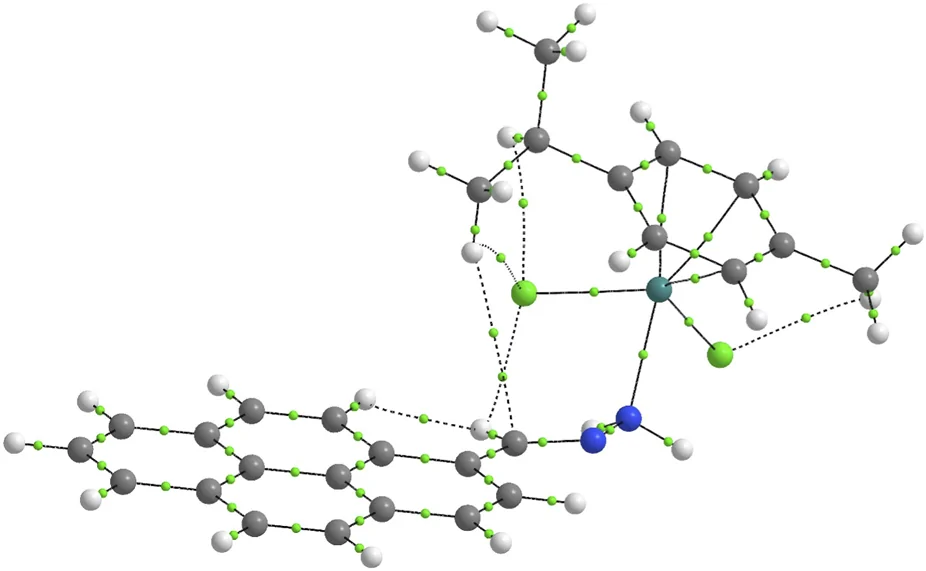
Bond Critical Points (BCPs, green dots) in the optimized structure of 1 (at B3LYP/6-311++G(d,p) (H,C,N,Cl)/LanL2DZ (Ru) level of theory).

The Quantum Theory of Atoms in Molecules is a method widely recognized for the examination of the interaction types and strength in transition metal complexes based on the properties of the bond critical points (Matveevskaya et al., 2022; Arlt, et al., 2021; Pallewela and Bettens, 2023; Sánchez-Coronilla et al., 2014). For the QTAIM analysis, several representative bonds were selected for further discussion and their density (ρ(r)), Laplacian (∇^2^ρ(r)), Lagrangian kinetic energy density (G(r)), potential energy density (V(r)), total energy density (H(r) = G(r) + V(r)), and interatomic bond energy (E_bond_ = V(r)/2) (Kasalović et al., 2024) are presented in Table 1. The last parameter is determined according to the Espinosa approach, as discussed in the reference (Espinosa et al., 1998). The main parameter for classifying interactions is the electron density. Electron densities exceeding 0.1 a. u. are common for charged (covalent) interactions, while van der Waals forces, ionic bonds, and hydrogen bonds have values around 0.01 a. u. (Lepetit et al., 2018; Soliman et al., 2017). Bonds can also be classified based on the ratio -G(r)/V(r), as proposed by Bianchi and coworkers (Bianchi et al., 2000). For covalent interactions, the value of this ratio is below 1; ionic interactions are characterized by a value above 2, while values between 1 and 2 denote partially covalent bonds. The total electron density (H(r)) and its sign can be used to distinguish between various interactions. Negative values of H(r) usually represent the covalent nature of the interaction. The BCPs in the structure of **1** are visualized in Figure 3, and the most important ligand-central metal ion interactions together with non-covalent interactions are represented in Table 1.

**TABLE 1:** The calculated Bond Critical Points (BCP) properties at the B3LYP/6-311++G(d,p) (H,C,N,Cl)/LanL2DZ (Ru) level of theory of **1**.

Bond	ρ(r) [a.u.]	∇^2^ρ(r) [a.u.]	G(r) [kJ mol^−1^]	V(r) [kJ mol^−1^]	H(r) [kJ mol^−1^]	G(r)/V (r)	E_bond_ [kJ mol^−1^]
Ru−C1	0.0758	0.235	204.1	−253.6	−49.5	0.8	−126.8
Ru−C2	0.0782	0.218	199.6	−256.3	−56.7	0.8	−128.2
Ru−C3	0.0794	0.221	201.4	−257.8	−56.4	0.8	−128.9
Ru−Cl1	0.0631	0.178	149.6	−182.4	−32.8	0.8	−91.2
Ru−Cl2	0.0649	0.181	153.0	−187.3	−34.3	0.8	−93.6
Ru−NH_2_	0.0770	0.315	236.6	−266.4	−29.8	0.9	−133.2
H2CH_*p*-cymen_∙∙∙CH	0.0024	0.008	3.8	−2.7	1.1	1.4	−1.4
H2CH_*p*-cymen_∙∙∙Cl1	0.0042	0.014	7.2	−5.5	1.8	1.3	−2.7
CH_*p*-cymen_∙∙∙Cl1	0.0070	0.024	12.6	−9.8	2.9	1.3	−4.9
CH∙∙∙Cl1	0.0077	0.023	12.4	−9.8	2.6	1.3	−4.9
H2CH_*p*-cymen∙∙∙_Cl2	0.0072	0.023	12.5	−9.7	2.8	1.3	−4.9

Analysis of the BCP parameters indicates that the strongest interactions are between Ru(II) and the *p*-cymene moiety. This is expected, as this part of the molecule originates from the starting salt. The electron densities and Laplacians are in a narrow range of 0.076–0.079 a. u. and 0.218–0.235 a. u., respectively. The total energies are negative, ranging from −49.5 to −56.7 kJ mol^−1^, indicating partial covalent character for these interactions. A similar result was reported for a Ru(II) complex with a diphenylmethylidenehydrazine ligand (Perendija et al., 2025). Additionally, the ratio of G(r) to V(r) for these interactions is less than 1. The interaction energies are almost equal, between −126.8 and −128.9 kJ mol^−1^. The differences in the values are a consequence of the geometry, which is not flexible, but in real systems, these values are expected to be equal. The properties of interactions between chlorido ligands and the central metal ion also depend on the other interactions that ligands form with neighboring groups. The electron densities and Laplacians are around 0.064 and 0.180 a. u., respectively. These interactions can also be considered as partially covalent with–G(r)/V(r) below 1, and negative total energy (−32.8 and −34.3 kJ mol^−1^). The difference of around 2 kJ mol^−1^ between the two Ru−Cl interaction energies is a consequence of the other interactions, as previously mentioned and depicted in Figure 3. The strongest interaction in the case of **1** is found between the central metal ion and the nitrogen atom of the hydrazine ligand. This interaction is characterized by the high electron density (0.077 a. u.) and Laplacian (0.315 a. u.). The partial covalent character is evident, as the total energy is negative (−29.8 kJ mol^−1^) and -G(r)/V(r) is lower than 1. The highest interaction energy of −133.2 kJ mol^−1^ is a consequence of the strong interaction between the lone pair of the nitrogen atom and the ruthenium (II) central metal ion. The delocalization within the hydrazine structure is limited, as a carbon atom lies between the aromatic system and the two nitrogen atoms, which influences the localization of the free electron pair on nitrogen.

In the examined structure, five interactions can be classified as weak. Three of these interactions involve the chlorido ligand in the vicinity of the *p*-cymene CH group. The interaction between the chlorido ligand and the C−H group has a low electron density of 0.008 a. u. and a Laplacian of 0.023 a. u. The total energy is positive (2.6 kJ mol^−1^), and the ratio between -G(r) and V(r) is greater than 1, which signifies the intermediate region between closed- and open-shell interactions. Three interactions with energies of −2.7 and −4.9 kJ mol^−1^ are present between the hydrogen atoms of the *p*-cymene moiety and the chlorido ligands. The weakest interaction (−1.4 kJ mol^−1^) is found between two hydrogen atoms of *p*-cymene and pyrene.

The results of second-order perturbation theory were also examined to support the previous findings, and the list of stabilization interactions and energies is presented in Table 2. Only the most notable interactions were selected for the discussion. The stabilization interactions within the structures of pyrene and *p*-cymene are omitted, although their importance for the overall stabilization of the system should be emphasized. When various constituents of the complex compound are concerned, the most numerous interactions are formed between *p*-cymene and the Ru(II) ion. More than 15 of these interactions, denoted as π(C−C) → LP*(Ru), have interaction energies between 59 and 447 kJ mol^−1^. Another type of interaction is formed between π(C−C) and σ*(Ru−Cl), with energies between 48 and 142 kJ mol^−1^. On the other side, there are interactions in which LP(Ru) acts as a donor, and π*(C−C) are acceptors. Two of these interactions have energies of 31 and 263 kJ mol^−1^. As previously mentioned, the strongest interaction was between the lone pair of the hydrazine nitrogen atom (LP(N)) and LP*(Ru), with a stabilization energy of 158 kJ mol^−1^. The hydrazine group as a whole is stabilizing the central metal ion through σ(N−N) → LP*(Ru) (179 and 280 kJ mol^−1^). The N−H group also influences the Ru(II) ion in the interactions marked as σ(N−H) → LP*(Ru) with energies between 56 and 180 kJ mol^−1^. Chlorido ligands are also influencing the Ru(II) ion through LP(Cl) → LP*(Ru) (42 and 113 kJ mol^−1^), which is consistent with previous QTAIM findings, in which the interactions between Ru(II) and chlorido ligands were weaker compared to *p*-cymene and hydrazine groups. The stabilization of the complex also involves interactions between the lone pair on Ru(II) and the C=C bonds of pyrene, with stabilization energies of around 10 kJ mol^−1^.

**TABLE 2:** The second-order perturbation stabilization energies obtained for the structure of **1**, optimized at the B3LYP/6-311++G(d,p) (H,C,N,Cl)/LanL2DZ (Ru) level of theory.

Donor	Acceptor	Stabilization energy [kJ mol^−1^]
π(C−C)_*p*-cymene_	LP*(Ru)	59–447
π(C−C)_*p*-cymene_	σ*(Ru−Cl)	48–142
LP(Ru)	π*(C−C)_*p*-cymene_	31 and 263
LP(N)	LP*(Ru)	158
σ(N−N)	LP*(Ru)	179 and 280
σ(N−H)	LP*(Ru)	56–180
LP(Cl)	LP*(Ru)	42 and 113
LP(Ru)	π*(C−C)_pyrene_	10

### 3.4 Solution stability of 1

The solution stability of 1 was evaluated by UV–Vis spectroscopy over 72 h. The spectra recorded in PBS containing 10% DMSO are shown in Supplementary Figure S3. These conditions were selected to evaluate the solution stability of the complex in an aqueous DMSO/PBS medium and were not intended to reproduce the exact solvent composition used in the cell viability assays. A gradual decrease in absorbance was observed during incubation, while the position of the absorption maxima remained essentially unchanged during the first 6 h, indicating that the overall electronic structure of the complex was largely preserved during this period. More pronounced spectral changes were observed after 24 h, suggesting gradual transformation of the complex in solution. Such behavior is consistent with the gradual aquation of the Ru–Cl bonds, a characteristic feature of (arene) (chlorido)Ru(II) complexes, while no evidence for rapid decomposition or dissociation of the coordinated hydrazone ligand was observed within the investigated time frame (Dougan et al., 2006).

### 3.5 Experimental evaluation of protein binding affinity

Owing to its high ligand-binding capacity and pronounced conformational flexibility, HSA is extensively employed as a model protein for investigating protein–ligand interactions. The intrinsic fluorescence of HSA originates predominantly from a single tryptophan residue (Trp214) located in subdomain IIA, whose emission is highly sensitive to alterations in the surrounding microenvironment (Kompany-Zareh et al., 2022). Consequently, tryptophan fluorescence serves as a powerful spectroscopic probe for studying binding processes, as interactions of small molecules or metal complexes in the vicinity of Trp214 can induce measurable changes in fluorescence intensity and/or shifts in the emission maximum due to quenching.

Fluorescence quenching may proceed via different mechanisms, including static quenching, dynamic (collisional) quenching, or a combination of both. Analysis of the temperature dependence of the Stern–Volmer constant (K_SV_) provides valuable insight into the dominant quenching mechanism: an increase in K_SV_ with increasing temperature is indicative of dynamic quenching, which arises from enhanced molecular diffusion and collision frequency, whereas a decrease in K_SV_ suggests static quenching, associated with ground-state complex formation between the quencher and HSA. For data analysis, the intercept of the Stern–Volmer plots was fixed at unity to facilitate comparison and interpretation of the quenching behavior (Cao et al., 2018; Dimitrić Marković et al., 2025; Szymańska et al., 2021).

**FIGURE 4:**
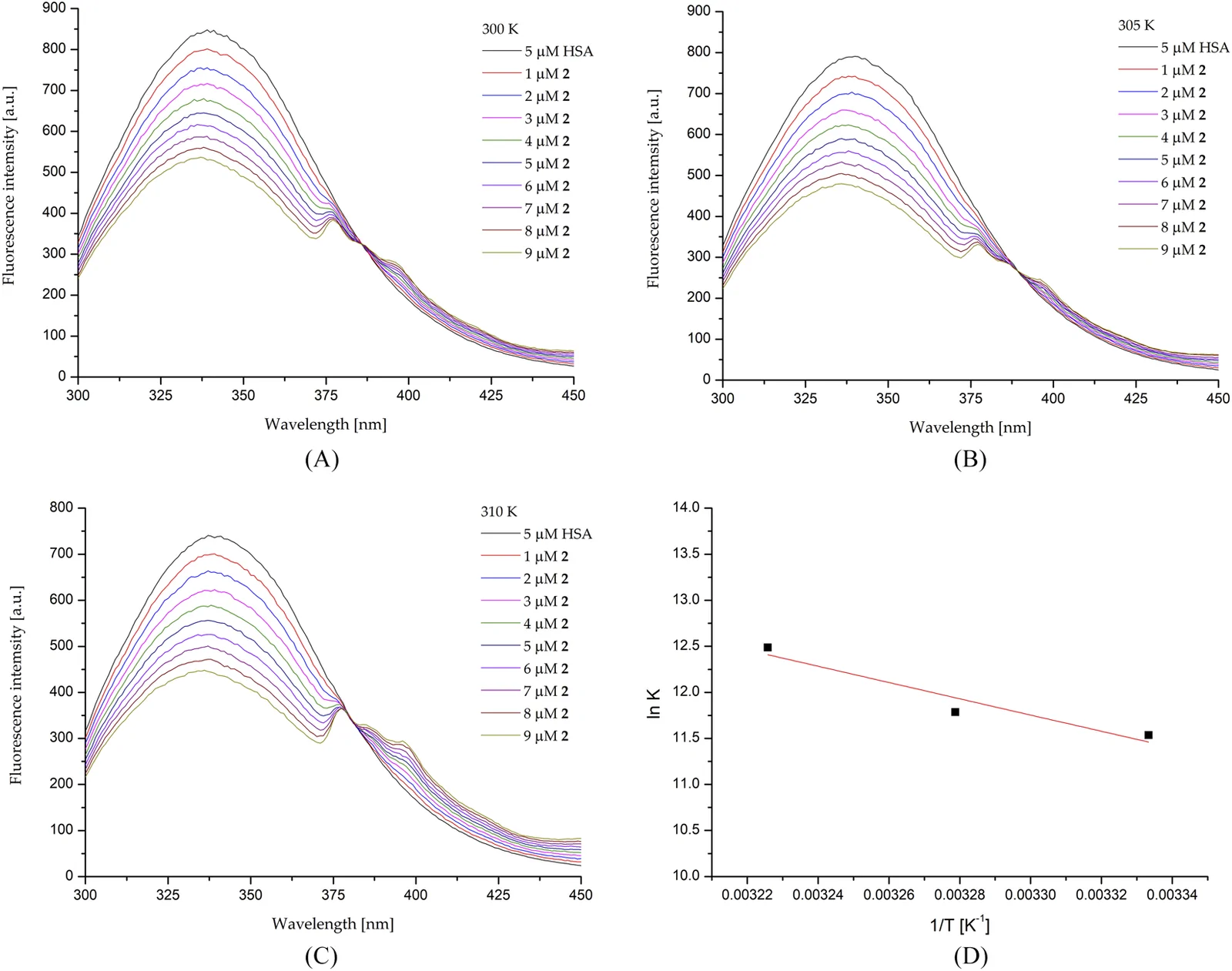
The fluorescence spectra of HSA after the addition of various concentrations of **1** at **(A)** 27 °C; **(B)** 32 °C; **(C)** 37 °C; and **(D)** the Van’t Hoff diagram for the binding process of 1 and HSA.

In the experimental procedure, HSA solutions were excited at 280 nm, a wavelength that efficiently excites both tryptophan and tyrosine residues, enabling sensitive monitoring of fluorescence changes upon ligand binding. Emission spectra were recorded at increasing concentrations of the complex and at three different temperatures. The resulting fluorescence spectra of HSA in the presence of varying concentrations of **1** are presented in Figure 4, while the corresponding binding and quenching parameters derived from the experimental data are summarized in Table 3.

**TABLE 3:** Stern–Volmer quenching constant, binding constant, the number of binding sites, ΔG_b_, ΔH_b_, and ΔS_b_.

T (K)	K_SV_ (M^−1^)	K_b_ (M^−1^)	*n*	ΔH_b_ (kJ/mol)	ΔS_b_ (J/molK)	ΔG_b_ (kJ/mol)	R^2^
300	6.34 × 10^4^	1.02 × 10^5^	1.04	73.4	340.1	−28.6	0,9256
305	6.98 × 10^4^	1.31 × 10^5^	1.05			−30.3	
310	7.46 × 10^4^	2.65 × 10^5^	1.11			−32.0	

Upon gradual addition of **1**, a pronounced decrease in the fluorescence emission intensity of HSA was observed, indicating effective quenching of the intrinsic tryptophan fluorescence. The isoemissive points were observed at 380.5, 385.5, and 388.5 nm, respectively (Figure 4). The Stern–Volmer quenching constant (K_SV_) values were found to range from 6.34 × 10^4^ to 7.46 × 10^4^ M^−1^ within the temperature interval of 300–310 K. As no systematic increase or decrease in KSV with temperature was observed, the results suggest the coexistence of both static and dynamic quenching mechanisms, implying simultaneous ground-state complex formation and collisional quenching.

The Stern–Volmer constant values obtained for complex **1** are higher than those reported for structurally related Ru(II) arene complexes with smaller aromatic ligands (K_SV_ ≈ 1.86–1.98 × 10^4^ M^−1^; Perendija et al., 2025). This enhanced quenching efficiency can be attributed to the presence of the pyrene moiety, which provides an extended π-conjugated system capable of strong π–π stacking interactions with aromatic residues of HSA, particularly Trp214.

The binding constant (K_b_) values are 1.02 × 10^5^ M^−1^ at 300 K, 1.31 × 10^5^ M^−1^ at 305 K, and 2.65 × 10^5^ M^−1^ at 310 K. For all temperatures examined, the binding stoichiometry parameter (n) ranged from 1.04 to 1.11, indicating that approximately one molecule of **1** binds to one molecule of HSA. The availability of binding data at three different temperatures enabled the reliable evaluation of the thermodynamic parameters governing the interaction.

The binding enthalpy change (ΔH_b_) was calculated to be 74.4 kJ mol^−1^, while the corresponding entropy change (ΔS_b_) was 340.1 J mol^−1^ K^−1^ (Table 3), revealing that the interaction is strongly endothermic and accompanied by a marked increase in system entropy. The Gibbs free energy change (ΔG_b_) was negative at all investigated temperatures, ranging from −28.6 to −32.0 kJ mol^−1^, thereby confirming the spontaneous nature of the binding process. The observed decrease in the magnitude of ΔG_b_ with increasing temperature suggests a slight strengthening of the interaction at higher temperatures. The obtained values of ΔG_b_ are comparable to those reported for the [RuCl_2_(η^6^-*p*-cymene) (bph-κN)] complex (Perendija et al., 2025).

The obtained thermodynamic parameters indicate that the binding of **1** to HSA is predominantly driven by hydrophobic interactions, with the binding process accompanied by the release of ordered water molecules from the hydrophobic surfaces of both the ligand and the protein, and additional contributions from van der Waals forces. This interpretation is consistent with the aromatic and hydrophobic character of the ligand framework, as well as with the simultaneous positive values of ΔH_b_ and ΔS_b_, which are characteristic of non-covalent, entropy-driven binding processes in protein–ligand systems.

### 3.6 Experimental evaluation of DNA binding affinity

#### 3.6.1 Spectrofluorimetric titration of **1** by CT-DNA

Understanding the interaction between the **1** and DNA constitutes a fundamental step in its biological assessment, as DNA binding is frequently linked to anticancer activity and potential applications in tumor therapy (Abdalla et al., 2023; Palchaudhuri and Hergenrother et al., 2007). In this regard, CT-DNA is widely used as a representative model to examine the DNA-binding behavior of small molecules and metal-based complexes. Detailed analysis of these interactions provides valuable insight into the possible mechanisms of action of DNA-targeted agents, including widely discussed binding modes such as intercalation between base pairs, minor- or major-groove association, and electrostatic interactions with the phosphate backbone. While native DNA exhibits only weak intrinsic fluorescence, its interaction with fluorescent ligands or emissive metal complexes can be efficiently monitored by spectrofluorimetric titration. Upon association of a metal complex with DNA, variations in the fluorescence intensity of either the complex or a DNA-bound probe may arise as a consequence of quenching or enhancement phenomena. Evaluation of these spectral changes enables the extraction of key binding parameters, including binding strength, stoichiometry, and the prevailing interaction mode. Notably, CT-DNA is particularly advantageous for such studies owing to its close structural similarity to human DNA and its well-characterized base composition.

**FIGURE 5:**
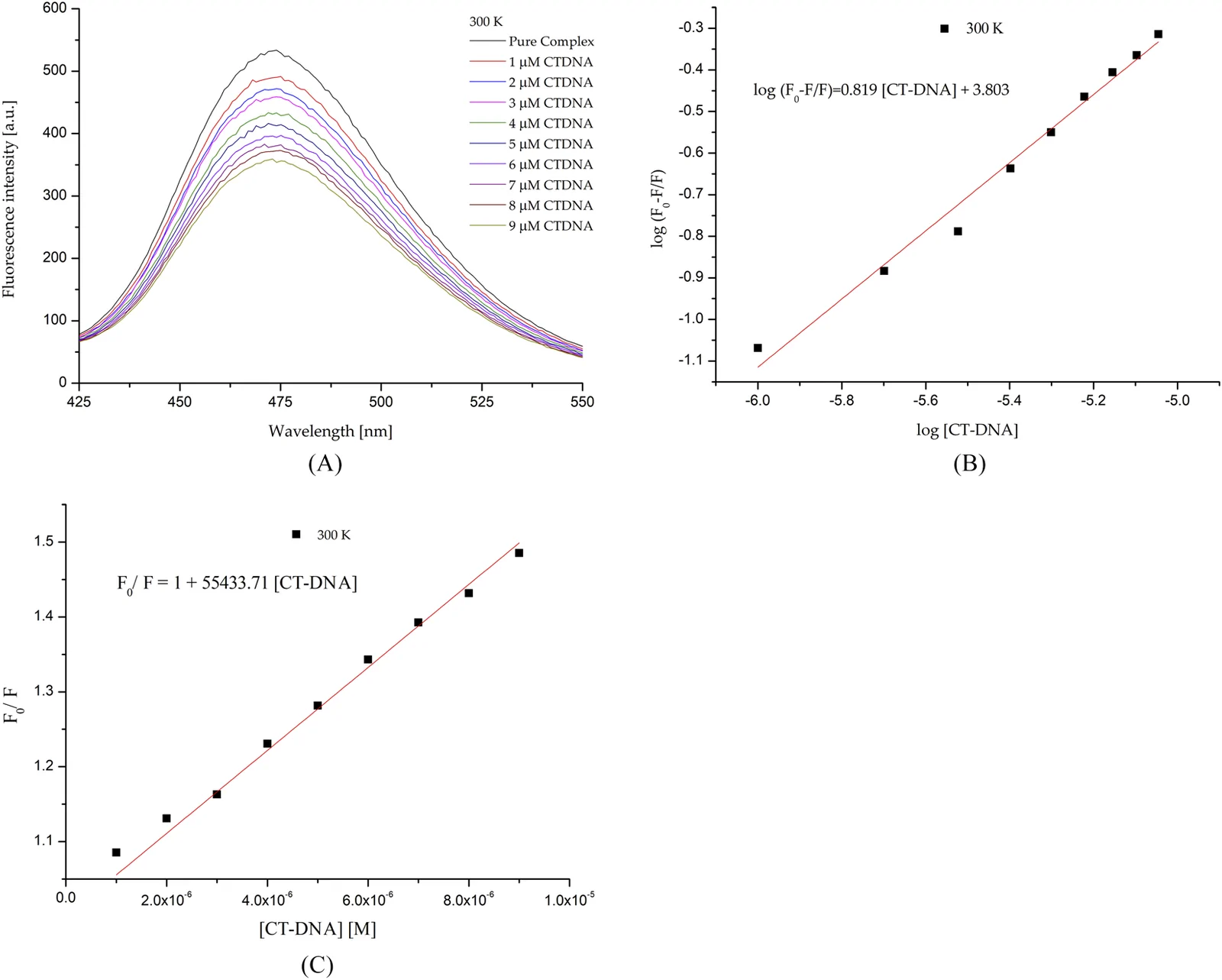
The fluorescence spectra of **1** upon interaction with various concentrations of calf thymus DNA (CT-DNA) at **(A)** 27 °C; **(B)** double-log SV diagram; **(C)** SV diagram.

Under the applied experimental conditions, the complex displayed a stable, intense fluorescence maximum at 473 nm upon excitation at 294 nm. Emission spectra were collected over 320–600 nm upon stepwise addition of CT-DNA, as previously reported (Perendija et al., 2025). The corresponding Stern–Volmer quenching constant (5.54 × 10^4^ M^−1^), binding constant (6.35 × 10^3^ M^−1^), Hill coefficient (0.82), and Gibbs free energy change at 300 K (−21.8 kJ mol^−1^) derived from the experimental data are shown in Figure 5. The value of the Gibbs free energy of binding indicates that binding is spontaneous.

#### 3.6.2 Competitive ethidium bromide displacement studies

The interaction between the synthesized complex and DNA was investigated using an ethidium bromide (EB) displacement assay. EB is commonly used as a fluorescent probe for studying DNA binding, since its fluorescence intensity increases markedly when it intercalates between base pairs of double-stranded DNA (Wilson et al., 2008). Upon binding to nucleic acids, EB forms stable complexes that exhibit an excitation wavelength around 520 nm and a fluorescence emission maximum near 600 nm. The enhancement of fluorescence originates from the insertion of the planar phenanthridinium chromophore between adjacent DNA base pairs within the double helix, a process known as intercalation (Psomas, 2008). Changes in fluorescence intensity, therefore, provide a convenient method for evaluating the interaction of other molecules or metal complexes with DNA through competitive displacement and fluorescence quenching (Zhao et al., 1998). In contrast, EB molecules that remain free in solution show very weak fluorescence because the excited state is efficiently quenched by solvent molecules (Dhar et al., 2005).

Supplementary Figure S4 presents the emission spectra of CT-DNA–EB in the presence of increasing concentrations of complex **1** at 27 °C. Progressive addition of the complex leads to a marked decrease in fluorescence intensity, consistent with partial displacement of the EB probe. From these data, the K_SV_ was determined to be 1.39 × 10^4^ M^−1^, while the binding constant derived from the double-log Stern–Volmer relationship was calculated as 1.15 × 10^4^ M^−1^. The binding stoichiometry parameter (n) was found to be approximately 0.98, suggesting that, on average, each molecule of the complex displaces one molecule of EB.

These results suggest that the synthesized complex interacts with DNA, leading to partial displacement of EB from its binding sites. Considering the extended planar pyrene aromatic system, an intercalative contribution appears plausible, however, groove-binding interactions cannot be excluded based on the present fluorescence data alone. The binding free energy, estimated from the binding constant, was calculated to be −23.3 kJ mol^−1^, indicating that the interaction is thermodynamically favorable under the applied experimental conditions.

Since the EB displacement assay provides indirect information on the binding mode, additional studies such as viscosity measurements, circular dichroism, or thermal denaturation experiments would be required to distinguish unambiguously between intercalative and groove-binding interactions. Nevertheless, the present results demonstrate that the complex is capable of binding to DNA and perturbing the interaction of EB with the double helix.

### 3.7 Molecular docking

#### 3.7.1 Binding affinity of **1** toward HSA, DNA, and oncogenic receptors

Molecular docking of **1** was performed to evaluate its binding affinity toward experimentally studied biomolecular targets (HSA and DNA) and six oncogenic receptors relevant to cancer cell survival and proliferation. The calculated binding energies (Table 4) revealed clear differences in target preference, with the BCL-2/BCL-xL protein showing the most favorable binding among all examined systems. The table summarizes the predicted binding free energies (ΔG, kJ mol^−1^) and binding constants (K_b_, μM) for **1** with the investigated receptors and DNA. Among all investigated targets, BCL-2/BCL-xL showed the most favorable binding energy and the lowest estimated Ki, indicating the highest predicted binding affinity.

**TABLE 4:** Calculated docking parameters for **1** toward HSA, DNA, and selected oncogenic targets.

Ruthenium complex – protein/DNA	ΔG_bind_	K_b_
1 – HSA	−27.7	13.95
1– DNA – intercalation	−21.1	201.1
1 – BCL-2/BCL-xL	−46.3	0.008
1 – JAK2	−45.5	0.011
1 – KRAS	−41.7	0.050
1 – BRAF	−40.3	0.088
1 – EGFR	−35.6	0.573
1 – BCR-ABL	−28.9	8.405

Particularly noteworthy is the close agreement between the calculated and experimentally determined binding energies for HSA and DNA, which supports the reliability of the docking protocol employed. For the **1**–HSA complex, the calculated binding free energy was −27.7 kJ mol^−1^, while the experimentally determined value was −28.6 kJ mol^−1^. The difference of only 0.9 kJ mol^−1^ indicates very good consistency between the computational and experimental approaches. Similarly, for the **1**–DNA complex, the theoretical value was −21.1 kJ mol^−1^, compared with the experimental value of −21.8 kJ mol^−1^, corresponding to a deviation of only 0.7 kJ mol^−1^. These small deviations between calculated and experimental binding free energies were used as internal validation metrics for the applied docking protocol. Such close agreement is particularly relevant because ruthenium complexes are challenging for conventional docking scoring functions due to their metal-centered coordination environment. Therefore, although the docking results should be interpreted as computational predictions, the agreement obtained for HSA and DNA supports the applicability of the docking protocol to the investigated systems.

According to the docking calculations, the binding affinity of **1** toward the investigated oncogenic targets followed the order: BCL-2/BCL-xL > JAK2 > KRAS > BRAF > EGFR > BCR-ABL. The most favorable binding was obtained for BCL-2/BCL-xL, with a calculated binding energy of −46.32 kJ/mol and an estimated binding constant of 0.008 μM, indicating very strong binding in the nanomolar range. A similarly high affinity was found for JAK2 (−45.48 kJ mol^−1^; K_i_ = 0.011 μM), while KRAS (−41.67 kJ mol^−1^; K_i_ = 0.050 μM) and BRAF (−40.25 kJ mol^−1^; K_i_ = 0.088 μM) also showed pronounced binding potential. In contrast, somewhat weaker but still relevant affinity was observed for EGFR, whereas BCR-ABL exhibited the lowest affinity among the tested oncogenic proteins. These findings suggest that **1** does not behave as a purely nonspecific binder, but rather displays a marked preference for structurally compatible and predominantly hydrophobic binding pockets, particularly those found in BCL-2/BCL-xL and JAK2. Importantly, the binding to BCL-2/BCL-xL was significantly more favorable than that calculated for HSA and DNA, indicating that although albumin binding and DNA interaction may contribute to transport and biological activity, the protein target may represent the dominant pharmacologically relevant binding site.

#### 3.7.2 Binding interactions with HSA

**FIGURE 6:**
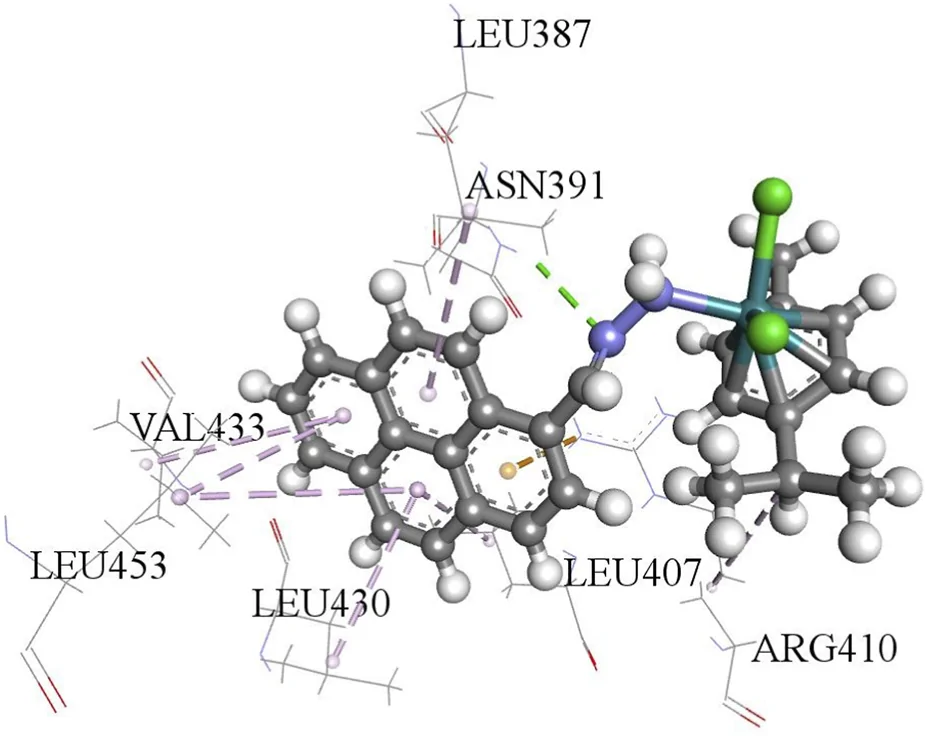
Hydrophobic (pink dotted lines) and hydrogen bond (green dotted lines) docking interactions of the most stable conformations of the selected compound with HSA.

Docking analysis of the **1**–HSA complex (Figure 6) showed that **1** binds within a largely hydrophobic environment, mainly formed by Leu387, Leu407, Leu430, Val433, and Leu453, with additional contributions from Arg410 and Asn391. The complex is stabilized predominantly by hydrophobic contacts involving the aromatic ligand framework, while a hydrogen bond with Asn391 further anchors the ligand within the binding pocket. This interaction pattern is consistent with the experimentally observed albumin affinity and supports the assumption that HSA may serve as an efficient transport protein for **1** in the bloodstream without excessively trapping the complex. The interaction of ruthenium complexes with HSA is often considered pharmacologically relevant because albumin can act as a transport reservoir, modulating distribution and bioavailability of metallodrugs (Liu et al., 2012; Dömötör et al., 2013; Dömötö and Enyedy 2019).

#### 3.7.3 Binding interactions with DNA

For the **1**–DNA complex (Supplementary Figure S5), the lowest-energy docking pose is consistent with an intercalative binding mode, in which the aromatic portion of the complex is positioned between adjacent DNA base pairs. The complex is stabilized predominantly through π–π stacking interactions with the nucleobases, while hydrogen bonding provides an additional contribution to binding stabilization. Although the calculated binding energy for DNA was less favorable than those obtained for the strongest protein targets, the good agreement between the theoretical and experimental ΔG values supports the feasibility of DNA binding.

Since molecular docking provides only a structural model of the complex–DNA interaction, additional biophysical studies are required to distinguish unambiguously between intercalative and groove-binding modes. Ruthenium polypyridyl and related complexes are well known to interact with DNA through intercalative or threading-intercalative binding modes, typically stabilized by aromatic stacking with base pairs (Hall et al., 2011; Cardin et al., 2017).

#### 3.7.4 Binding interactions with BCL-2/BCL-xL

Among the investigated proteins, **1** showed the most favorable predicted binding toward BCL-2/BCL-xL (Supplementary Figure S6). The binding mode indicates that **1** can be accommodated within the protein’s hydrophobic pocket, where the aromatic ligand scaffold establishes multiple hydrophobic interactions with residues such as Leu108, Ala149, and Val152. Additional stabilization is predicted to be provided by nearby polar residues, including Arg102, Asp107, and Glu153, and by at least one hydrogen bond that helps orient the complex within the binding site. This combination of extensive hydrophobic contacts and favorable polar interactions may explain the lowest calculated ΔG value and the smallest K_b_ among all investigated targets.

Given the well-established anti-apoptotic role of BCL-2/BCL-xL proteins, the predicted favorable binding of **1** suggests that it may interfere with apoptosis-related signaling pathways. Among the examined systems, the computational results indicate that BCL-2/BCL-xL may represent potential molecular targets of **1**, providing the rationale for their subsequent investigation using molecular dynamics simulations and MM/PBSA analysis.

#### 3.7.5 Molecular dynamics simulation

To further examine the stability of the most favorable docking complex, the 1–BCL-2/BCL-xL system was subjected to a 100 ns molecular dynamics simulation in explicit solvent. The MD analysis included RMSD, RMSF, radius of gyration (Rg), and MM/PBSA binding free energy calculations to evaluate the structural stability, flexibility, compactness, and energetic favorability of the complex over time. The time evolution of the backbone RMSD for the 1–BCL-2/BCL-xL complex is shown in Supplementary Figure S7. The RMSD remained within a relatively narrow range throughout the simulation, averaging 0.20 ± 0.03 nm, indicating that the complex reached a stable conformational state with minimal structural deviations. Although small fluctuations were observed during the trajectory, these remained within the expected range for a stable protein–ligand complex and did not suggest any substantial destabilization or ligand-induced unfolding of the protein. A slight increase in RMSD at later stages of the simulation likely reflects normal conformational adaptation of the protein to the bound ruthenium complex rather than loss of binding stability. The RMSD profile supports the conclusion that the docked pose remained stable during the 100 ns simulation.

The RMSF profile of the **1**–BCL-2/BCL-xL complex is presented in Supplementary Figure S8. The average RMSF value was 0.14 ± 0.06 nm, indicating generally low residue mobility across the protein. Most residues fluctuated within a limited range, suggesting that the protein framework remained stable during the simulation. Higher RMSF peaks were observed only for selected residue regions, which most likely correspond to flexible loop segments and solvent-exposed parts of the protein rather than the rigid core of the binding groove. Such localized fluctuations are common in MD simulations and do not indicate complex instability. On the contrary, the relatively low average RMSF suggests that ligand binding did not induce excessive local disorder and that the interaction with **1** was compatible with the structural integrity of the BCL-2/BCL-xL binding site.

The radius of gyration profile shown in Supplementary Figure S9 provides information on the protein’s compactness during the simulation. The average Rg value was 2.68 ± 0.01 nm, with only minor fluctuations over the 100 ns trajectory. This result indicates that the protein preserved a stable fold and did not undergo significant expansion or collapse upon ligand binding. The narrow distribution of Rg values further confirms that the **1**–BCL-2/BCL-xL complex remained structurally compact throughout the simulation, which is consistent with the RMSD and RMSF observations.

#### 3.7.6 MM/PBSA Binding free energy analysis

The thermodynamic stability of the complex was additionally evaluated using the MM/PBSA approach, and the results are summarized in Supplementary Table S2. The MM/PBSA analysis yielded a total binding free energy of −72.38 ± 14.7 kJ mol^−1^, confirming that the binding of **1** to BCL-2/BCL-xL is thermodynamically favorable. The complex formation was mainly stabilized by electrostatic (−331.1 ± 283.3 kJ mol^−1^) and van der Waals interactions (−106.3 ± 13.2 kJ mol^−1^), whereas the polar solvation term (378.4 ± 282.1 kJ mol^−1^) opposed binding. The nonpolar solvation contribution was modest but favorable (−13.5 ± 1.5 kJ mol^−1^), contributing to a negative binding free energy. Nevertheless, the favorable nonbonded interactions were sufficient to overcome the solvation penalty, yielding a net negative binding free energy.

The MM/PBSA results are consistent with the docking and molecular dynamics analyses. The stable RMSD profile, low average RMSF values, and nearly constant radius of gyration, Rg, indicate that the protein–ligand complex remained structurally stable throughout the simulation. Although the docking, molecular dynamics simulations, and MM/PBSA calculations consistently indicate favorable interactions of **1** with BCL-2/BCL-xL, the proposed role of these proteins as molecular targets is based on computational predictions. Experimental validation using appropriate biochemical and cellular assays will be required to confirm their involvement in the biological activity of the complex.

### 3.8 EPR measurements

**FIGURE 7:**
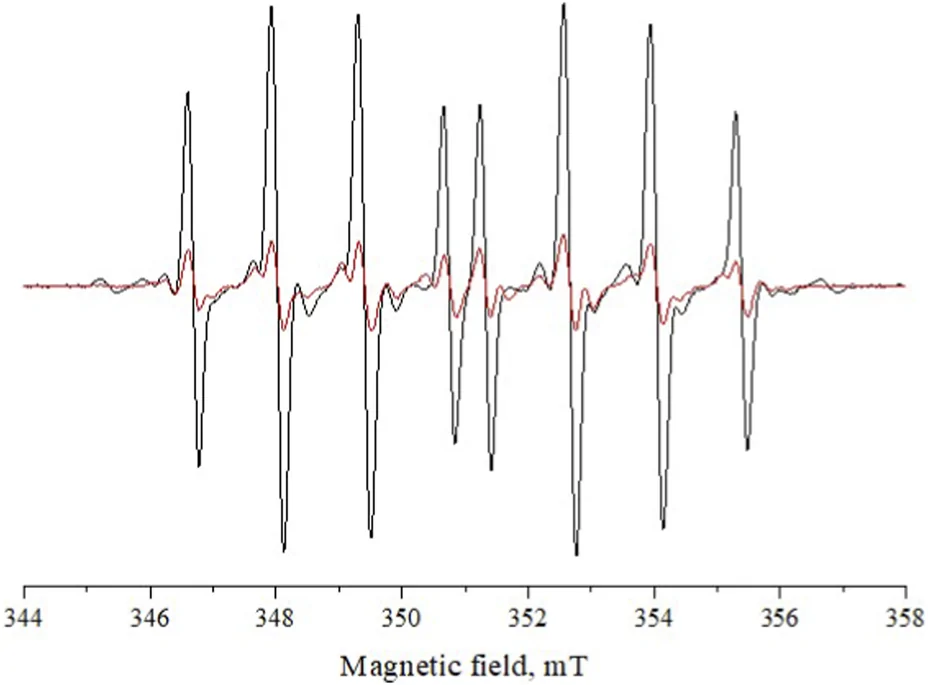
The EPR spectra of DEPMPO/OH recorded in the absence (control, black) and presence of the investigated **1** (red).

Depending on the radical species involved, the examined complex showed significantly different scavenging efficiencies for the same concentration, suggesting a strong selectivity in its antiradical behavior. There was very little activity (0.70%) against DPPH radicals. Because the DPPH assay involves interaction with a relatively bulky and sterically shielded radical, the very low inhibition suggests limited reactivity of the complex in this model system. One possible explanation is steric constraints that reduce the effective interaction between the complex and the DPPH radical. Furthermore, accessibility and reaction efficiency may be impacted by the lipophilic nature of DPPH and the mixed solvent system. Consequently, the antioxidant potential of this complex may not be sufficiently reflected by the DPPH assay. On the other hand, the complex showed a strong scavenging effect towards hydroxyl radicals (83.00%). Significant suppression of the corresponding EPR signal suggests effective attenuation of radical presence under the applied conditions for these highly reactive and non-selective species. This result suggests that the complex is particularly effective in systems involving highly reactive oxygen species. The EPR spectra illustrating the decrease of the DEPMPO/OH spin adduct signal are shown in Figure 7, as the **1** exhibited the most pronounced scavenging effect in this system, while spectra obtained for the other radical systems are shown in the Supplementary Figure S10. The activity toward superoxide anion radicals was moderate (12.60%). Superoxide is less reactive than hydroxyl radicals and often requires redox-active centers or superoxide dismutase mimics to be scavenged. The complex does not effectively compete with the spin trap for O_2_•^−^under the applied conditions, which is evidenced by the comparatively low inhibition. Similarly, limited activity was detected against the ascorbyl radical (14.67%). These results suggest that the complex interacts less pronouncedly with radical species that are more stable or less reactive. Variability in activity may be caused by variations in radical charge, size, stability, and reaction environment.

The results demonstrate that the antioxidant profile of the complex is not generalized but radical-specific. The strong effect observed in the reaction with the hydroxyl radical, combined with low or modest activity in the other assays, highlights the importance of using multiple radical models when evaluating the antiradical properties of compounds.

### 3.9 *In vitro* cytotoxic and oxidative stress–related effects of 1 in cancer cell lines

The biological activity profile of **1** revealed a pronounced and selective anticancere effect toward melanoma (A-375) and hepatocellular carcinoma (HCC) cells, whereas marginal or no effects were observed in breast cancer (MDA-MB-231), pancreatic cancer (MIA PaCa-2), leukemia (HEL), and chronic myeloid leukemia (K-562) cells (Supplementary Figure S11). The MTT assay demonstrated a clear concentration-dependent reduction in cell viability in A-375 and HCC cells, with IC50 concentrations of 233.6 ± 24.8 and 94.2 ± 19.1 μM, respectively (Supplementary Figure S11). In contrast, the remaining cell lines maintained more than 60% viability even at concentrations higher than 500 μM (Supplementary Figure S11), indicating substantially lower sensitivity to treatment with **1**. Such differential responsiveness suggests that the cytotoxic action of complex **1** may depend on tumor-specific metabolic or redox characteristics.

Although the anticancer activity of 1 is low to moderate compared with several recently reported Ru(II)-based anticancer complexes, direct comparison of IC_50_ values should be interpreted with caution because the biological activity of ruthenium complexes strongly depends on ligand structure, lipophilicity, intracellular accumulation, and molecular targets. Recent Ru(II)-dithiocarbazate complexes bearing triphenylphosphine ligands exhibit low micromolar cytotoxicity, which has been attributed to enhanced lipophilicity, mitochondrial accumulation, ROS overproduction, and apoptosis induction rather than to DNA damage alone (Sahu et al., 2023). Similarly, Ru(II)-arene complexes containing lipophilic phosphine, phosphite, or arsine ligands display considerably higher cytotoxicity than their PTA-containing analogues, demonstrating the important role of ligand design in determining biological activity (Kljun et al., 2023). Comprehensive studies on Ru-based metallodrugs have further shown that their therapeutic potential should not be evaluated solely on the basis of cytotoxic potency, since many representative compounds, including RAPTA derivatives and KP1019-related systems, exert their biological effects through multiple mechanisms involving oxidative stress, mitochondrial dysfunction, apoptosis, protein interactions, and modulation of signaling pathways (Lee et al., 2020).

In this context, comparison with structurally related Ru(II)-arene hydrazone complexes is particularly informative. Our previously reported analogues containing hydrazone or hydrazine ligands exhibited higher cytotoxic activity than **1** (Eichhorn et al., 2023; Eichhorn et al., 2024), indicating that relatively small structural modifications of the coordinated hydrazone ligand can markedly influence the biological properties of this class of compounds. Replacement of these ligands by the more extended pyrene aromatic system is expected to alter the physicochemical properties of the complex, such as its lipophilicity, solution behavior, protein binding, and cellular uptake, thereby influencing its biological activity. The lower cytotoxicity of **1** most likely reflects these structural differences, further illustrating the pronounced structure–activity relationship within this class of Ru(II)-arene complexes. To further elucidate the mechanism underlying the observed anticancer activity, markers of oxidative stress were analyzed. Lipid peroxidation, assessed by malondialdehyde (MDA) formation, was significantly elevated in A-375 cells following treatment with complex **1**, whereas no statistically significant increase was detected in HCC cells (Supplementary Figure S12). Specifically, MDA levels increased more than twofold in A-375 cells compared with untreated controls (Supplementary Figure S12), indicating extensive oxidative damage to membrane lipids. Since MDA is a well-established end product of polyunsaturated fatty acid oxidation, its accumulation strongly suggests enhanced reactive oxygen species (ROS) generation following exposure of A-375 cells with **1**. These findings are consistent with previous reports showing that many ruthenium (II)-based anticancer agents induce oxidative stress as an important component of their cytotoxic mechanism (Bhattacharya et al., 2025; Garrosa-Miró et al., 2025).

Protein oxidation was evaluated through the determination of protein carbonyl (PC) content. Treatment with complex **1** significantly increased PC levels in both A-375 and HCC cells (Supplementary Figure S13), indicating that proteins represent an important target of oxidative injury. Interestingly, the increase was particularly pronounced in A-375 cells, where protein carbonylation more than doubled relative to controls (Supplementary Figure S13). Although HCC cells also exhibited a significant elevation in PC levels, the magnitude of the increase was lower than that observed in melanoma cells (Supplementary Figure S13). The simultaneous increase in both MDA and PC levels in A-375 cells (Supplementary Figures S12 and S13) suggests widespread oxidative damage affecting multiple cellular macromolecules, whereas in HCC cells, oxidative injury appears to be predominantly directed toward proteins rather than membrane lipids. Such differences may reflect variations in antioxidant defense systems, membrane composition, or cellular metabolism between the two tumor cell types.

The ability of oxidative stress to trigger cell death pathways has been extensively documented (Liu et al., 2025; Saxena, Welsh, and He, 2024). Excessive ROS production can impair mitochondrial function, induce DNA damage, oxidize proteins and lipids, and ultimately activate apoptotic or necrotic signaling cascades. Therefore, the observed increase in oxidative stress biomarkers (Supplementary Figures S12 and S13) provides a plausible explanation for the marked reduction in viability detected in A-375 and HCC cells (Supplementary Figure S11). Moreover, the stronger oxidative response observed in melanoma cells correlates well with their slightly higher sensitivity to the treatment with **1** (Supplementary Figures S11 and S13), supporting the hypothesis that ROS-mediated cytotoxicity contributes substantially to the anticancer activity of complex **1**.

Because tumor progression depends not only on cell proliferation but also on invasive potential, the effects of complex **1** on matrix metalloproteinases were investigated. Matrix metalloproteinase-2 (MMP-2) activity showed a modest increase in A-375 cells but was significantly reduced in HCC cells (Supplementary Figure S14A). In contrast, MMP-9 activity was not significantly altered in either cell line despite a tendency toward reduced activity in A-375 cells and increased activity in HCC cells (Supplementary Figure S14B). MMP-2 and MMP-9 are gelatinases involved in extracellular matrix degradation and are frequently associated with tumor invasion, metastasis, and angiogenesis. The significant reduction of MMP-2 activity in HCC cells (Supplementary Figure S14A), therefore, suggests that complex **1** may possess anti-invasive properties in addition to its direct cytotoxic effects. The lack of significant changes in MMP-9 activity (Supplementary Figure S14B) indicates that the modulation of metalloproteinase pathways by this compound may be selective and cell-type dependent.

The observed decrease in MMP-2 activity in HCC cells (Supplementary Figure S14A) may also be linked to oxidative stress. ROS can regulate metalloproteinase expression and activation through multiple signaling pathways, including MAPK, NF-κB, and AP-1 (Baek et al., 2024; Shin et al., 2025; Veljkovic et al., 2025). Depending on the cellular context and intensity of oxidative stimulation, ROS may either activate or suppress MMP production. The concomitant increase in protein oxidation (Supplementary Figure S13) and reduction in MMP-2 activity (Supplementary Figure S14A) observed in HCC cells suggest that oxidative damage induced by complex **1** may interfere with signaling pathways required for metalloproteinase activation, thereby limiting the invasive phenotype of these cells.

The present results indicate that the anticancer activity of Ru(II) complex **1** is associated with the induction of oxidative stress and, in HCC cells, with suppression of MMP-2 activity. The strong cytotoxicity observed in melanoma and hepatocellular carcinoma cells (Supplementary Figure S11) correlates with elevated levels of oxidative damage markers (Supplementary Figures S12 and S13), supporting a ROS-mediated mechanism of cellular injury. Furthermore, the reduction of MMP-2 activity in HCC cells (Supplementary Figure S14A) suggests a potential role of **1** in inhibiting tumor invasiveness. These findings highlight the therapeutic potential of Ru(II)-based compounds as multifunctional anticancer agents that simultaneously impair tumor cell survival and modulate processes associated with tumor progression. Further studies examining ROS generation, mitochondrial dysfunction, apoptosis-related pathways, and metastatic behavior will be necessary to fully elucidate the molecular mechanisms responsible for the biological effects of **1**. Since the primary objective of the present study was the synthesis, structural characterization, theoretical investigation, and preliminary biological evaluation of a new (arene)Ru(II) complex, assessment of its selectivity using non-malignant cell lines was beyond the scope of this work and will be addressed in future studies.

The pronounced hydroxyl radical scavenging activity observed in the cell-free EPR experiments does not contradict the increased oxidative stress detected in cancer cells following treatment with **1**, as the observations originate from fundamentally different biological contexts. The cell-free EPR experiment measures the direct chemical reaction of **1** with hydroxyl radicals, whereas intracellular oxidative stress arises from complex cellular processes involving mitochondrial function, antioxidant defenses, and disruption of redox homeostasis. Such dual redox behavior has been widely reported for transition-metal complexes which may act as radical scavengers in simple chemical systems while simultaneously promoting oxidative stress in cancer cells through perturbation of intracellular redox balance (Murillo et al., 2022). Accordingly, efficient hydroxyl radical scavenging under cell-free conditions does not necessarily prevent intracellular ROS accumulation. The increased lipid peroxidation and protein oxidation observed herein therefore most likely reflect indirect ROS production resulting from disruption of cellular redox homeostasis rather than direct formation of hydroxyl radicals by the complex.

## 4 Conclusion

A new Ru(II) half-sandwich arene complex, **1**, containing the hydrazone ligand (*E*)-1-(pyren-1-ylmethylidene)hydrazine, was successfully synthesized and characterized by FTIR, NMR, elemental analysis, and DFT calculations. The good agreement between the experimental and theoretical data supported the proposed pseudo-octahedral piano-stool arrangement, while QTAIM analysis indicated that partially covalent Ru–N, Ru–Cl, and Ru–arene interactions contribute to its stability.

Complex **1** interacted with both human serum albumin and calf thymus DNA. The experimental binding studies, together with molecular docking, indicate that the complex is capable of binding DNA, while computational analyses identified BCL-2/BCL-xL as a plausible molecular target. Molecular dynamics simulations and MM/PBSA calculations further supported the stability of this interaction.

The complex showed selective radical-scavenging activity, exhibiting the highest activity toward hydroxyl radicals. *In vitro* studies demonstrated moderate to low but selective cytotoxic activity toward melanoma and hepatocellular carcinoma cells, accompanied by increased lipid and protein oxidation. These findings indicate that **1** possesses biologically relevant protein- and DNA-binding properties together with moderate anticancer activity, providing a basis for further investigation of this class of (arene)Ru(II) complexes.

## Data Availability

The original contributions presented in the study are included in the article/Supplementary Material, further inquiries can be directed to the corresponding authors.

## References

[B1] Abdalla E. M. Al-Sulami A. I. Aly S. A. Abd-Allah M. T. Nasr G. M. Albohy S. A. H. (2023). Synthesis, characterization, DNA binding, DFT, anticancer, antibacterial, and the effect of gamma irradiation of novel Co(II), Ag (I), and Cd (II) complexes with hydrazone derivatives. J. Saudi Chem. Soc. 27, 101770. 10.1016/j.jscs.2023.101770

[B83] Abraham M. J. Murtola T. Schulz R. Páll S. Smith J. C. Hess B. (2015). GROMACS: High performance molecular simulations through multi-level parallelism from laptops to supercomputers. SoftwareX, 1–2, 19–25. 10.1016/j.softx.2015.06.001

[B2] Alessio E. (2017). Thirty years of the drug candidate NAMI‐A and the myths in the field of ruthenium anticancer compounds: a personal perspective. Eur. J. Inorg. Chem. 2017, 1549–1560. 10.1002/ejic.201600986

[B3] Alessio E. Messori L. (2019). NAMI-A and KP1019/1339, two iconic ruthenium anticancer drug candidates Face-to-Face: a case story in medicinal inorganic chemistry. Molecules 24, 1995. 10.3390/molecules24101995 31137659 PMC6571951

[B5] Arlt S. Petković V. Ludwig G. Eichhorn T. Lang H. Rüffer T. (2021). Arene Ruthenium(II) complexes bearing the κ-P or κ-P,κ-S Ph2P(CH2)3SPh ligand. Molecules 26, 1860. 10.3390/molecules26071860 33806101 PMC8036862

[B84] Arora R. Linders J. T. M. Aci-Seche S. Verheyen T. Van Heerde E. Brehmer D. (2023). Design, synthesis and characterisation of a novel type II B-RAF paradox breaker inhibitor. Eur. J. Med. Chem. 250, 115231. 10.1016/j.ejmech.2023.115231 36878151

[B6] Bader R. F. W. (1985). Atoms in molecules. Acc. Chem. Res. 18, 9–15. 10.1021/ar00109a003

[B7] Bader R. F. W. Slee T. S. Cremer D. Kraka E. (1983). Description of conjugation and hyperconjugation in terms of electron distributions. J. Am. Chem. Soc. 105, 5061–5068. 10.1021/ja00353a035

[B8] Baek J. Kim J. Lee H. Park S. Kim Y. (2024). 1-Kestose blocks UVB-Induced skin inflammation and photoaging by suppressing ROS-Mediated MAPK/AP-1 signaling and matrix metalloproteinase expression. Nutrients 16, 1587. 10.3390/nu16091587 38892519

[B9] Baker A. Aguirre-Hernández C. Halldén G. Parker A. (2018). Designer oncolytic adenovirus: coming of Age. Cancers 10, 201. 10.3390/cancers10060201 29904022 PMC6025169

[B85] Baskić D. D. Radosavljević G. Čokanović V. Jeftić I. Zelen I. Popović S. (2005). Serumski nivoi NO, IL-18 i MDA kod pacijenata sa karcinomom dojke. Medicus 6, 62–65.

[B10] Becke A. D. (1993). Density-functional thermochemistry. III. The role of exact exchange. J. Chem. Phys. 98, 5648–5652. 10.1063/1.464913

[B11] Bergamo A. Masi A. Jakupec M. A. Keppler B. K. Sava G. (2009). Inhibitory effects of the ruthenium complex KP1019 in models of mammary cancer cell migration and invasion. Met.-Based Drugs 2009, 1–9. 10.1155/2009/681270 19789639 PMC2748298

[B12] Bhattacharya S. Adon T. Dsouza K. Kumar H. Y. (2025). Exploring the future of metal‐based anticancer agents: a comprehensive review of ruthenium‐based complexes. Chem. Sel. 10, e202404147. 10.1002/slct.202404147

[B13] Bianchi R. Gervasio G. Marabello D. (2000). Experimental electron density analysis of Mn_2_ (CO)_10_: metal−metal and Metal−Ligand bond characterization. Inorg. Chem. 39, 2360–2366. 10.1021/ic991316e 12526497

[B86] O’Boyle N. M. Banck M. James C. A. Morley C. Vandermeersch T. Hutchison G. R. (2011). Open babel: An open chemical toolbox. J. Cheminform. 3, 33. 10.1186/1758-2946-3-33 21982300 PMC3198950

[B14] Bohmann J. A. Weinhold F. Farrar T. C. (1997). Natural chemical shielding analysis of nuclear magnetic resonance shielding tensors from gauge-including atomic orbital calculations. J. Chem. Phys. 107, 1173–1184. 10.1063/1.474464

[B87] Dömötör O. Hartinger C. G. Bytzek A. K. Kiss T. Keppler B. K. adn Enyedy E. A. (2013). Characterization of the binding sites of the anticancer ruthenium (III) complexes KP1019 and KP1339 on human serum albumin via competition studies. J. Biol. Inorg. Chem. 18 (1), 9–17. 10.1007/s00775-012-0944-6 23076343

[B88] Dömötör O. Enyedy É. A. (2019). Binding mechanisms of half-sandwich Rh (III) and Ru (II) arene complexes on human serum albumin: a comparative study. J. Biol. Inorg. Chem. 24 (5), 703–719. 10.1007/s00775-019-01683-0 31300922 PMC6682546

[B15] Brabec V. Nováková O. (2006). DNA binding mode of ruthenium complexes and relationship to tumor cell toxicity. Drug resist. updat. 9, 111–122. 10.1016/j.drup.2006.05.002 16790363

[B16] Cao X. He Y. Liu D. He Y. Hou X. Cheng Y. (2018). Characterization of interaction between scoparone and bovine serum albumin: spectroscopic and molecular docking methods. RSC Adv. 8, 25519–25525. 10.1039/C8RA04065F 35539773 PMC9082657

[B89] Cardin C. J. Kelly J. M. Quinn S. J. (2017). Photochemically active DNA-intercalating ruthenium and related complexes–insights by combining crystallography and transient spectroscopy. Chem. Sci. 8 (7), 4705–4723. 10.1039/C7SC01070B 28936338 PMC5596416

[B90] Cheng H. Nair S. K. Murray B. W. Almaden C. Bailey S. Baxi S. (2016). Discovery of PF- 06459988, a potent WT-sparing, irreversible EGFR mutant inhibitor (T790M). J. Med. Chem. 59, 2005–2024. 26756222 10.1021/acs.jmedchem.5b01633

[B18] Crabtree R. H. (2009). The Organometallic Chemistry of the Transition Metals. 5th Edn. Hoboken, New York: Wiley.

[B19] Dasmahapatra U. Maiti B. Alam M. M. Chanda K. (2024). Anti-cancer property and DNA binding interaction of first row transition metal complexes: a decade update. Eur. J. Med. Chem. 275, 116603. 10.1016/j.ejmech.2024.116603 38936150

[B189] Dunning T. H. , (1989). Gaussian basis sets for use in correlated molecular calculations. I. The atoms boron through neon and hydrogen. J. Chem. Phys. 90(2), 1007–1023. 10.1063/1.456153

[B21] Dhar S. Nethaji M. Chakravarty A. R. (2005). Effect of charge transfer bands on the photo-induced DNA cleavage activity of [1-(2-thiazolylazo)-2-naphtholato]copper(II) complexes. J. Inorg. Biochem. 99, 805–812. 10.1016/j.jinorgbio.2004.12.014 15708802

[B22] Dimitrić Marković J. Dimić D. Eichhorn T. Milenković D. Pavićević A. Đikić D. (2025). Ru(II) complexes with 3,4-Dimethylphenylhydrazine: exploring *in vitro* anticancer activity and protein affinities. Biomolecules 15, 350. 10.3390/biom15030350 40149886 PMC11940238

[B23] Dougan S. J. Melchart M. Habtemariam A. Parsons S. Sadler P. J. (2006). Phenylazo-pyridine and phenylazo-pyrazole chlorido Ruthenium(II) arene complexes: Arene loss, aquation, and cancer cell cytotoxicity. Inorg. Chem. 45, 10882–10894. 10.1021/ic061460h 17173447

[B91] Dunning T. H. , (1989). Gaussian basis sets for use in correlated molecular calculations. I. The atoms boron through neon and hydrogen. J. Chem. Phys. 90(2), 1007–1023. 10.1063/1.456153

[B26] Eichhorn T. Dimić D. D. Marković Z. Kaluđerović G. N. (2023). Synthesis, spectroscopic characterization and DFT analysis of dichlorido(η^6^-p-cymene)ruthenium(II) complexes with isonicotinate-polyethylene glycol ester ligands. J. Serb. Chem. Soc. 88 (12), 1335–1354. 10.2298/JSC230412070E

[B27] Eichhorn T. Đošić M. Dimić D. Morgan I. Milenković D. Rennert R. (2024). Ru(II)‐Nitrophenylhydrazine/Chlorophenylhydrazine complexes: nanoarchitectonics, biological evaluation and *in silico* study. Eur. J. Inorg. Chem. 27, e202300683. 10.1002/ejic.202300683

[B28] Espinosa E. Molins E. Lecomte C. (1998). Hydrogen bond strengths revealed by topological analyses of experimentally observed electron densities. Chem. Phys. Lett. 285, 170–173. 10.1016/S0009-2614(98)00036-0

[B30] Foster J. P. Weinhold F. (1980). Natural hybrid orbitals. J. Am. Chem. Soc. 102, 7211–7218. 10.1021/ja00544a007

[B31] Frisch M. J. Trucks G. W. Schlegel H. B. Scuseria G. E. Robb M. A. Cheeseman J. R. (2009). Gaussian 09, Revision D.01. Wallingford, CT: Gaussian, Inc.

[B32] Garrosa-Miró Y. Muñoz-Moreno L. D'Errico G. Tancredi M. Carmena M. J. Ottaviani M. F. (2025). Ruthenium(II) and copper(II) polyamine complexes as promising antitumor agents: synthesis, characterization, and biological evaluation. Dalton Trans. 54, 8298–8314. 10.1039/D4DT03377A 40232207

[B33] Gasser G. Metzler-Nolte N. (2012). The potential of organometallic complexes in medicinal chemistry. Curr. Opin. Chem. Biol. 16, 84–91. 10.1016/j.cbpa.2012.01.013 22366385

[B34] Gatter K. C. Brown G. Trowbridge I. S. Woolston R. E. Mason D. Y. (1983). Transferrin receptors in human tissues: their distribution and possible clinical relevance. J. Clin. Pathol. 36, 539–545. 10.1136/jcp.36.5.539 6302135 PMC498283

[B92] Ghuman J. Zunszain P. A. Petitpas I. Bhattacharya A. A. Otagiri M. Curry S. (2005). Structural basis of the drug-binding specificity of human serum albumin. J. Mol. Biol. 353, 38–52. 10.1016/j.jmb.2005.07.075 16169013

[B35] Glavinić U. Nakarada Đ. Stevanović J. Gašić U. Ristanić M. Mojović M. (2025). Chemical composition and antioxidant activity of Prokupac Grape Pomace extract: implications for redox modulation in Honey Bee cells. Antioxidants 14, 751. 10.3390/antiox14060751 40563383 PMC12189821

[B36] Gulaczyk I. Kręglewski M. Valentin A. (2003). The N–N stretching band of hydrazine. J. Mol. Spectrosc. 220, 132–136. 10.1016/S0022-2852(03)00106-1

[B37] Hay P. J. Wadt W. R. (1985). *Ab initio* effective core potentials for molecular calculations. Potentials for K to Au including the outermost core orbitals. J. Chem. Phys. 82, 299–310. 10.1063/1.448975

[B93] Hess B. Bekker H. Berendsen H. J. C. Fraaije J. G. E. M. (1997). LINCS: a Linear constraint solver for molecular simulations. J. Comput. Chem. 18(12), 1463–1472. 10.1002/(SICI)1096-987X(199709)18:123.0.CO;2-H

[B38] Hongthong K. Nhukeaw T. Temboot P. Dyson P. J. Ratanaphan A. (2021). Anticancer activity of RAPTA-EA1 in triple-negative BRCA1 proficient breast cancer cells: single and combined treatment with the PARP inhibitor olaparib. Heliyon 7, e07749. 10.1016/j.heliyon.2021.e07749 34430738 PMC8371217

[B94] Hunter J. C. Manandhar A. Carrasco M. A. Gurbani D. Gondi S. Westover K. D. (2015). Biochemical and structural analysis of common cancer-associated KRAS mutations. Mol. Cancer Res. 13, 1325–1335. 10.1158/1541-7786.MCR-15-0203 26037647

[B39] Jabłońska-Wawrzycka A. Rogala P. Michałkiewicz S. Hodorowicz M. Barszcz B. (2013). Ruthenium complexes in different oxidation states: synthesis, crystal structure, spectra and redox properties. Dalton Trans. 42, 6092–6101. 10.1039/c3dt32214a 23381742

[B40] Jelisić S. Stanimirović Z. Ristanić M. Nakarada Đ. Mojović M. Bošnjaković D. (2024). The potential of Agaricus bisporus in mitigating pesticide-induced oxidative stress in honey bees infected with Nosema ceranae. Life 14, 1498. 10.3390/life14111498 39598296 PMC11595567

[B41] Kasalović M. P. Jelača S. Milanović Ž. Maksimović-Ivanić D. Mijatović S. Lađarević J. (2024). Novel triphenyltin(iv) compounds with carboxylato N -functionalized 2-quinolones as promising potential anticancer drug candidates: *in vitro* and *in vivo* evaluation. Dalton Trans. 53, 8298–8314. 10.1039/D4DT00182F 38661529

[B42] Keith T. A. (2016). Aimall. Overland Park, KS: TK Gristmill Software. Version 16.05.18.

[B43] Kenny R. G. Marmion C. J. (2019). Toward multi-targeted platinum and ruthenium Drugs-A new paradigm in cancer drug treatment regimens? Chem. Rev. 119, 1058–1137. 10.1021/acs.chemrev.8b00271 30640441

[B44] Kljun J. Rebernik M. Balsa L. M. Kladnik J. Rapuš U. Trobec T. (2023). Exploring pta alternatives in the development of ruthenium–arene anticancer compounds. Molecules 28, 2499. 10.3390/molecules28062499 36985471 PMC10058425

[B45] Kompany-Zareh M. Akbarian S. Najafpour M. M. (2022). Unsupervised recognition of components from the interaction of BSA with Fe cluster in different conditions utilizing 2D fluorescence spectroscopy. Sci. Rep. 12, 16875. 10.1038/s41598-022-20768-6 36207446 PMC9547014

[B46] Kong C. Chen X. (2022). Combined photodynamic and photothermal therapy and immunotherapy for cancer treatment: a review. Int. J. Nanomed. 17, 6427–6446. 10.2147/IJN.S388996 36540374 PMC9760263

[B47] Lalrempuia R. Kollipara M. R. Carroll P. J. (2003). Syntheses and characterization of arene ruthenium (II) complexes containing N,N′-donor Schiff base ligands. Crystal and molecular structure of [(η^6^-C_6_Me_6_)Ru(C_5_H_4_N-2-CH N C_6_H_4_-p-NO_2_)]PF_6_ . Polyhedron 22, 605–609. 10.1016/S0277-5387(02)01430-4

[B48] Lee S. Y. Kim C. Y. Nam T. G. (2020). Ruthenium complexes as anticancer agents:a brief history and perspectives. Drug Des. Dev. Ther. 14, 5375–5392. 10.2147/DDDT.S275007 33299303 PMC7721113

[B49] Leijen S. Burgers S. A. Baas P. Pluim D. Tibben M. Van Werkhoven E. (2015). Phase I/II study with ruthenium compound NAMI-A and gemcitabine in patients with non-small cell lung cancer after first line therapy. Invest. New Drugs 33, 201–214. 10.1007/s10637-014-0179-1 25344453

[B50] Lepetit C. Vabre B. Canac Y. Alikhani M. E. Zargarian D. (2018). Pentacoordinated, square pyramidal cationic PCP Ni(II) pincer complexes: ELF and QTAIM topological analyses of nickel–triflate interactions. Theor. Chem. Acc. 137, 141. 10.1007/s00214-018-2332-y

[B95] Levine R. L. Garland D. Oliver C. N. Amici A. Climent I. Lenz A. G. (1990). Determinationof carbonyl content in oxidatively modified proteins. Methods Enzymol. 186, 464–478. 10.1016/0076-6879(90)86141-h 1978225

[B96] Li P. Merz K. M. Jr. (2016). MCPB.py: A Python-based metal center parameter builder. J. Chem. Inf. Model. 56(4), 599–604. 10.1021/acs.jcim.5b00674 26913476

[B51] Liang J. Li H. Huang K. Ning D. Yan F. Chen W. (2024). Pyrene-1-carboxaldehyde hydrazone modified mesoporous silica foam-based strategy for efficient and accurate electroanalysis of copper in food samples. J. Food Compos. Anal. 134, 106587. 10.1016/j.jfca.2024.106587

[B52] Lilge L. Roufaiel M. Lazic S. Kaspler P. Munegowda M. A. Nitz M. (2020). Evaluation of a Ruthenium coordination complex as photosensitizer for PDT of bladder cancer: cellular response, tissue selectivity and *in vivo* response. Transl. Biophot. 2, e201900032. 10.1002/tbio.201900032

[B97] Ling S. P. Ming L. C. Dhaliwal J. S. Gupta M. Ardianto C. Goh K. W. (2022). Role of immunotherapy in the treatment of cancer: A systematic review, Cancers (Basel) 14(21), 5205. 10.3390/cancers14215205 36358624 PMC9655090

[B98] Lipscomb L. A. Peek M. E. Zhou F. X. Bertrand J. A. VanDerveer D. Williams L. D. (1994). Water ring structure at DNA interfaces: Hydration and dynamics of DNA-anthracycline complexes. Biochemistry, 33, 3649– 3659. 10.1021/bi00178a023 8142363

[B53] Liu S. Liu J. Wang Y. Deng F. Deng Z. (2025). Oxidative stress: signaling pathways, biological functions, and disease. MedComm 6 (7), e70268. 10.1002/mco2.70268 40599237 PMC12209598

[B100] Liu Y. Yu Q. Wang C. Sun D. Huang Y. Zhou Y. (2012). Ruthenium (II) complexes binding to human serum albumin and inducing apoptosis of tumor cells. Inorg. Chem. Commun. 24, 104–109. 10.1016/j.inoche.2012.08.009

[B54] Lv M. Zheng Y. Dai X. Zhao J. Hu G. Ren M. (2024). Ruthenium(ii)–Arene complex triggers immunogenic ferroptosis for reversing drug resistance. J. Med. Chem. 67, 20156–20171. 10.1021/acs.jmedchem.4c01467 39312756

[B55] Marenich A. V. Cramer C. J. Truhlar D. G. (2009). Universal solvation model based on Solute electron density and on a continuum model of the solvent defined by the bulk dielectric constant and atomic surface tensions. J. Phys. Chem. B 113, 6378–6396. 10.1021/jp810292n 19366259

[B56] Matveevskaya V. V. Pavlov D. I. Samsonenko D. G. Bonfili L. Cuccioloni M. Benassi E. (2022). Arene-ruthenium(II) complexes with tetracyclic oxime derivatives: synthesis, structure and antiproliferative activity against human breast cancer cells. Inorg. Chim. Acta 535, 120879. 10.1016/j.ica.2022.120879

[B99] Miao Y. Virtanen A. Zmajkovic J. Hilpert M. Skoda R. C. Silvennoinen O. (2024). Functional and structural characterization of clinical-stage Janus kinase 2 inhibitors identifies determinants for drug selectivity. J. Med. Chem. 67, 10012–10024. 10.1021/acs.jmedchem.4c00197 38843875 PMC11215726

[B57] Milosavljević M. Nakarada Đ. Gostiljac M. Marković J. D. Jevtović V. Rakić A. (2025). Structural and biological properties of isoproterenol, comparison with epinephrine and norepinephrine. J. Chem. Sci. 137, 48. 10.1007/s12039-025-02381-y

[B200] Miller B. R. McGee T. D. Swails J. M. Homeyer N. Gohlke H. Roitberg A. E. (2012). MMPBSA.py: An efficient program for end-state free energy calculations. J. Chem. Theory Comput. 8, 1532–1541. 10.1021/ct300418h 26605738

[B101] Morris G. M. Huey R. Lindstrom W. Sanner M. F. Belew R. K. Goodsell D. S. (2009). AutoDock4 and AutoDockTools4: Automated docking with selective receptor flexibility. J. Comput. Chem. 30(16), 2785–2791. 10.1002/jcc.21256 19399780 PMC2760638

[B102] Mosmann T. , (1983) Rapid colorimetric assay for cellular growth and survival: application to proliferation and cytotoxicity assays. J. Immunol. Methods 65(1-2), 55–63. 10.1016/0022-1759(83)90303-4 6606682

[B58] Murillo M. I. Gaiddon C. Le Lagadec R. (2022). Targeting of the intracellular redox balance by metal complexes towards anticancer therapy. Front. Chem. 10, 967337. 10.3389/fchem.2022.967337 36034648 PMC9405673

[B103] Nguyen W. Lee E. F. Evangelista M. Lee M. Harris T. J. Colman P. M. (2021). Optimization of Benzothiazole and thiazole hydrazones as inhibitors of schistosome BCL-2. ACS Infect. Dis. 7, 1143–1163. 10.1021/acsinfecdis.0c00700 33523649

[B59] Palchaudhuri R. Hergenrother P. J. (2007). DNA as a target for anticancer compounds: methods to determine the mode of binding and the mechanism of action. Curr. Opin. Biot. 18, 497–503. 10.1016/j.copbio.2007.09.006 17988854

[B60] Pallewela G. N. Bettens R. P. A. (2023). A DFT, TDDFT and QTAIM study of the acridine pincer ligand-based Ru(II) and Rh(III) complexes: detailed analysis of the metal-F bonding. Chem. Pap. 77, 47–61. 10.1007/s11696-022-02340-8

[B62] Perendija S. Dimić D. Eichhorn T. Rakić A. Saso L. Nakarada Đ. (2025). Synthesis, characterization, HSA/DNA binding, and cytotoxic activity of [RuCl_2_(η^6^-p-cymene)(bph-κN)] complex. Molecules 30, 3088. 10.3390/molecules30153088 40807263 PMC12348660

[B63] Pettinari R. Marchetti F. Condello F. Pettinari C. Lupidi G. Scopelliti R. (2014). Ruthenium(II)–Arene RAPTA type complexes containing curcumin and bisdemethoxycurcumin display potent and selective anticancer activity. Organometallics 33, 3709–3715. 10.1021/om500317b

[B64] Pham T. Roth S. Kong J. Guerra G. Narasimhan V. Pereira L. (2018). An update on immunotherapy for solid tumors: a review. Ann. Surg. Oncol. 25, 3404–3412. 10.1245/s10434-018-6658-4 30039324

[B65] Psomas G. (2008). Mononuclear metal complexes with ciprofloxacin: synthesis, characterization and DNA-binding properties. J. Inorg. Biochem. 102, 1798–1811. 10.1016/j.jinorgbio.2008.05.012 18621421

[B104] Reckel S. Gehin C. Tardivon D. Georgeon S. Kukenshoner T. Lohr F. (2017). Structural and functional dissection of the DH and PH domains of oncogenic Bcr-Abl tyrosine kinase. Nat. Commun. 8, 2101. 10.1038/s41467-017-02313-6 29235475 PMC5727386

[B66] Reedijk B. J. (2008). Metal-Ligand Exchange kinetics in platinum and ruthenium complexes: significance for effectiveness as anticancer drugs. Platin. Met. Rev. 52, 2–11. 10.1595/147106708X255987

[B67] Sahu G. Patra S. A. Lima S. Das S. Gorls H. Plass W. (2023). Ruthenium(II)-Dithiocarbazates as anticancer Agents:Synthesis, solution behavior, and Mitochondria-Targeted apoptotic cell death. Chem. Eur. J. 29, e202202694. 10.1002/chem.202202694 36598160

[B68] Sánchez-Coronilla A. Sánchez-Márquez J. Zorrilla D. Martín E. I. De Los Santos D. M. Navas J. (2014). Convergent study of Ru–ligand interactions through QTAIM, ELF, NBO molecular descriptors and TDDFT analysis of organometallic dyes. Mol. Phys. 112, 2063–2077. 10.1080/00268976.2014.884729

[B105] Santos-Martins D. Tillack A. F. Eberhardt J. Forli S. (2021). AutoDock-GPU: GPU-accelerated docking. J. Chem. Inf. Model., 61(2), 293–304. 10.1021/acs.jcim.1c00203 PMC10683950 34278794

[B69] Saxena R. Welsh C. M. He Y.-W. (2024). Targeting regulated cell death pathways in cancers for effective treatment: a comprehensive review. Front. Cell Dev. Biol. 12, 1462339. 10.3389/fcell.2024.1462339 39620145 PMC11604647

[B70] Shin M. H. Kwon Y. Lee S. Kim J. Park H. Choi Y. (2025). Protective effect of a novel metal-phenolic network composite against ultraviolet-induced skin damage by modulating MAPK/AP-1/NF-κB signaling pathways and attenuating oxidative stress in human keratinocytes. Biomed. Pharmacother. 186, 118874. 10.1016/j.biopha.2025.118874 41352006

[B106] Simović A. R. Milenković D. Šeklić D. Jovanović M. Milović E. Međedović M. (2025). Exploring biginelli hybrids in the AI-driven development of ruthenium complexes: Anticancer activity, DNA/HSA binding study, impacts on apoptosis and BCL-2/BCL-XL suppression. J. Inorg. Biochem. 272, 112988. 10.1016/j.jinorgbio.2025.112988 40644785

[B71] Soliman S. M. Albering J. Abu-Youssef M. A. M. (2017). Structural analyses of two new highly distorted octahedral copper(II) complexes with quinoline-type ligands; Hirshfeld, AIM and NBO studies. Polyhedron 127, 36–50. 10.1016/j.poly.2017.01.051

[B72] Strasser S. Pump E. Fischer R. C. Slugovc C. (2015). On the chloride lability in electron-rich second-generation ruthenium benzylidene complexes. Monatsh. Chem. 146, 1143–1151. 10.1007/s00706-015-1484-x

[B73] Süss-Fink G. (2010). Areneruthenium complexes as anticancer agents. Dalton Trans. 39, 1673–1688. 10.1039/B916860P 20449402

[B74] Swaminathan S. Karvembu R. (2023). Dichloro Ru(II)-p-cymene-1,3,5-triaza-7-phosphaadamantane (RAPTA-C): a case study. ACS Pharmacol. Transl. Sci. 6, 982–996. 10.1021/acsptsci.3c00085 37470017 PMC10353064

[B75] Szymańska M. Pospieszna-Markiewicz I. Mańka M. Insińska-Rak M. Dutkiewicz G. Patroniak V. (2021). Synthesis and spectroscopic investigations of schiff base ligand and its bimetallic Ag(I) complex as DNA and BSA binders. Biomolecules 11, 1449. 10.3390/biom11101449 34680081 PMC8533391

[B76] Thota S. Rodrigues D. A. Crans D. C. Barreiro E. J. (2018). Ru(II) compounds: Next-Generation anticancer metallotherapeutics? J. Med. Chem. 61, 5805–5821. 10.1021/acs.jmedchem.7b01689 29446940

[B107] Valdés-Tresanco M. E. Valdés-Tresanco M. S. Valiente P. A. Moreno E. (2021). gmx_MMPBSA: a new tool to perform end-state free energy calculations with GROMACS. J. Chem. Theory Comput. 17, 6281–6291. 10.1021/acs.jctc.1c00645 34586825

[B77] Veljkovic A. Stanojevic G. Brankovic B. Roumeliotis S. Leivaditis K. Djordjevic B. (2025). MMP-9 activation via ROS/NF-κB signaling in colorectal cancer progression: molecular insights and prognostic–therapeutic perspectives. Cell. Mol. Biol. 47 (7), 557. 10.14715/cmb/2025.71.7.557 PMC12293130 40729026

[B78] Wang W. Wang L. Zhang Y. Shi Y. Zhang R. Chen L. (2024). Chiral iridium-based TLD-1433 analogues: exploration of enantiomer-dependent behavior in photodynamic cancer therapy. Inorg. Chem. 63, 7792–7798. 10.1021/acs.inorgchem.4c00215 38619892

[B108] Wilson W. D. Tanious F. A. Mathis A. Tevis D. Hall J. E. Boykin D. W. (2008). Antiparasitic compounds that target DNA. Biochimie 90, 999–1014. 10.1016/j.biochi.2008.02.017 18343228 PMC2515095

[B109] Zhang Y. Forli S. Omelchenko A. Sanner M. F. (2019). AutoGridFR: improvements on AutoDock affinity maps and associated software tools. J. Comput. Chem. 40(32), 2882–2886. 10.1002/jcc.26054 31436329 PMC7737998

[B81] Zhang C. Kang T. Wang X. Song J. Zhang J. Li G. (2022). Stimuli-responsive platinum and ruthenium complexes for lung cancer therapy. Front. Pharmacol. 13, 1035217. 10.3389/fphar.2022.1035217 36324675 PMC9618881

[B82] Zhao G. Lin H. Zhu S. Sun H. Chen Y. (1998). Dinuclear palladium(II) complexes containing two monofunctional [Pd(en)(pyridine)Cl]^+^ units bridged by Se or S. Synthesis, characterization, cytotoxicity and kinetic studies of DNA-binding. J. Inorg. Biochem. 70, 219–226. 10.1016/S0162-0134(98)10019-3 9720307

