# Supplementary material for: A novel Ru(II) complex with pyrene-1-carbaldehyde hydrazone: structural, electronic, theoretical, and biological studies
Source: Front Chem. 2026 Aug 31;14:1904958. doi: 10.3389/fchem.2026.1904958 (PMC13572611; doi:10.3389/fchem.2026.1904958)
Supplement: Supplementary file 1 [file DataSheet1.pdf]

Supplementary Material

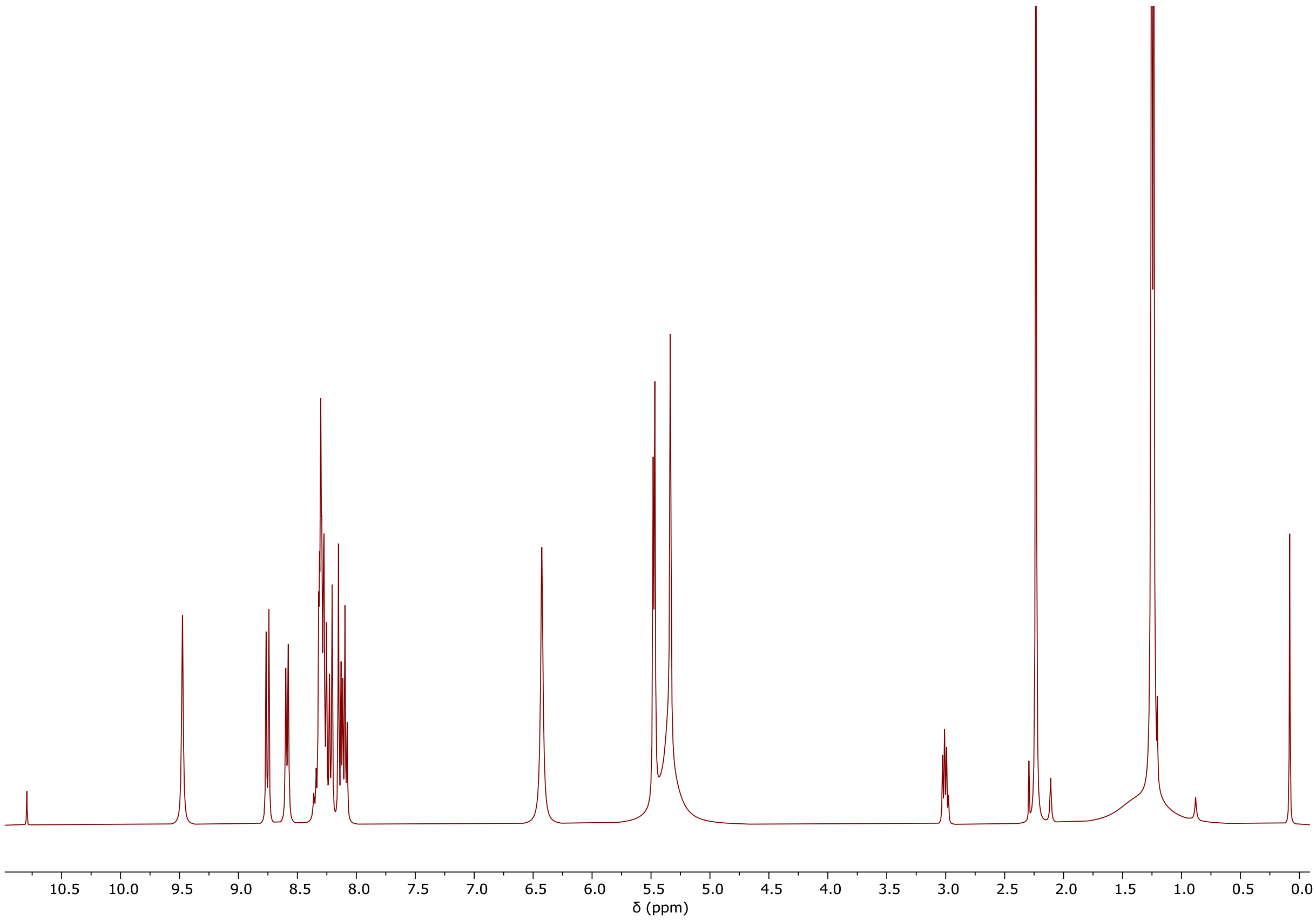

Supplementary Figure 1. ^{1}H NMR spectrum of **1** (DCM-d_2, 400 MHz)

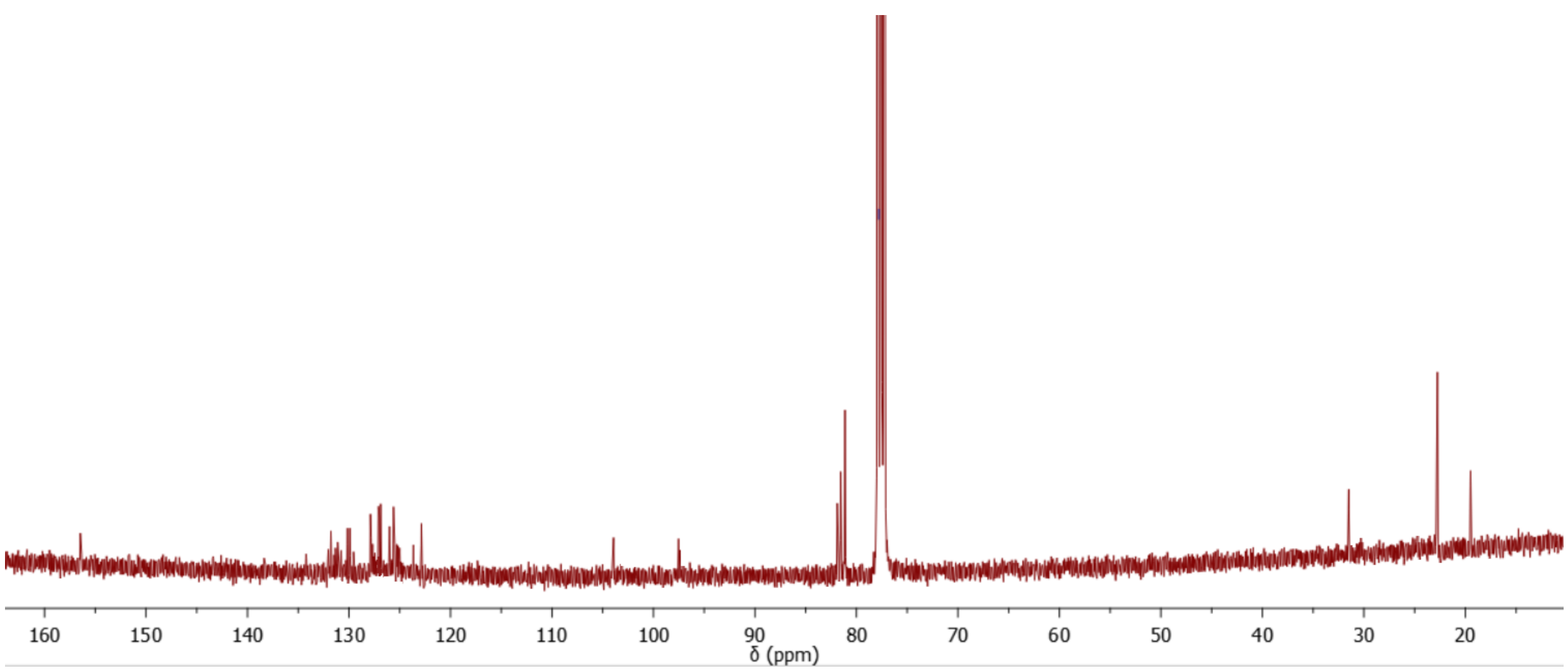

Supplementary Figure 2. ^{13}C NMR spectrum of **1** ($CDCl_3$, 100 MHz).

Supplementary Table 1. Experimental and theoretical ^{1}H NMR and ^{13}C NMR chemical shifts calculated at B3LYP/6-311++G(d,p)(H,C,N,Cl)/LanL2DZ(Ru) level of theory.

**^{1}H NMR**			**^{13}C NMR**		
H atom	δ(exp.) [ppm]	δ(calc.) [ppm]	C atom	δ(exp.) [ppm]	δ(calc.) [ppm]
$C(CH_3)_2$	1.24	1.26	CH_3, *p*-cym	17.6, 22.4, 22.8	18.1, 18.8, 21.8
CCH_3	2.23	2.65	$CH(CH_3)_2$, *p*-cym	31.3	35.7
C*H*	3.00	3.03	C_{Ar}, η^6-*p*-cym	73.0, 79.9, 81.3, 88.3, 99.1, 112.0	83.9, 88.2, 90.3, 93.5, 104.8, 116.3
$CHCH_{cym}$	5.38	5.13	C_{Ar}, pyrene	123.0, 124.8, 125.3, 125.9, 126.5, 126.9, 127.1, 127.2, 129.3, 130.0, 130.7, 131.4, 132.2	124.9, 128.2, 128.7, 128.8, 129.3, 129.5, 131.0 ,131.3, 132.1, 132.5, 134.9, 135.1, 135.9, 136.3, 136.4, 140.1
N*H*	5.50	5.41	H*C*=N	158.4	159.5
CH_{Pyr}	8.11	8.48			
CH_{Pyr}	8.28	8.60			
CH_{Pyr}	8.59	8.69			
CH_{Pyr}	8.75	8.89			
CH_{Pyr}	9.48	9.50			
CH_{Pyr}	10.80	9.79			
R		0.9965			0.9830
MAE [ppm]		0.14			6.85

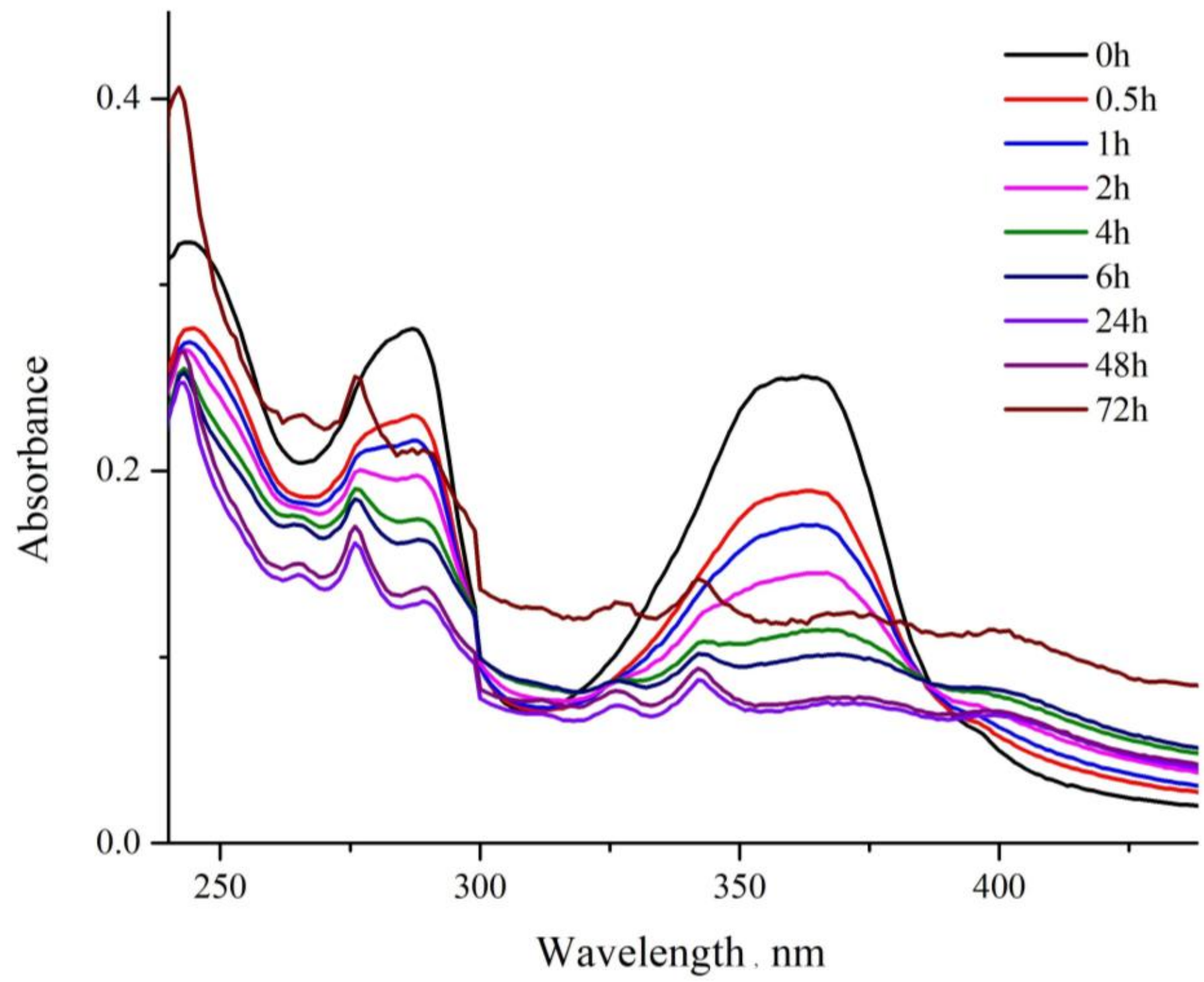

Supplementary Figure 3. Time-dependent UV–Vis absorption spectra of **1** recorded in PBS containing 10% DMSO over 72 h.

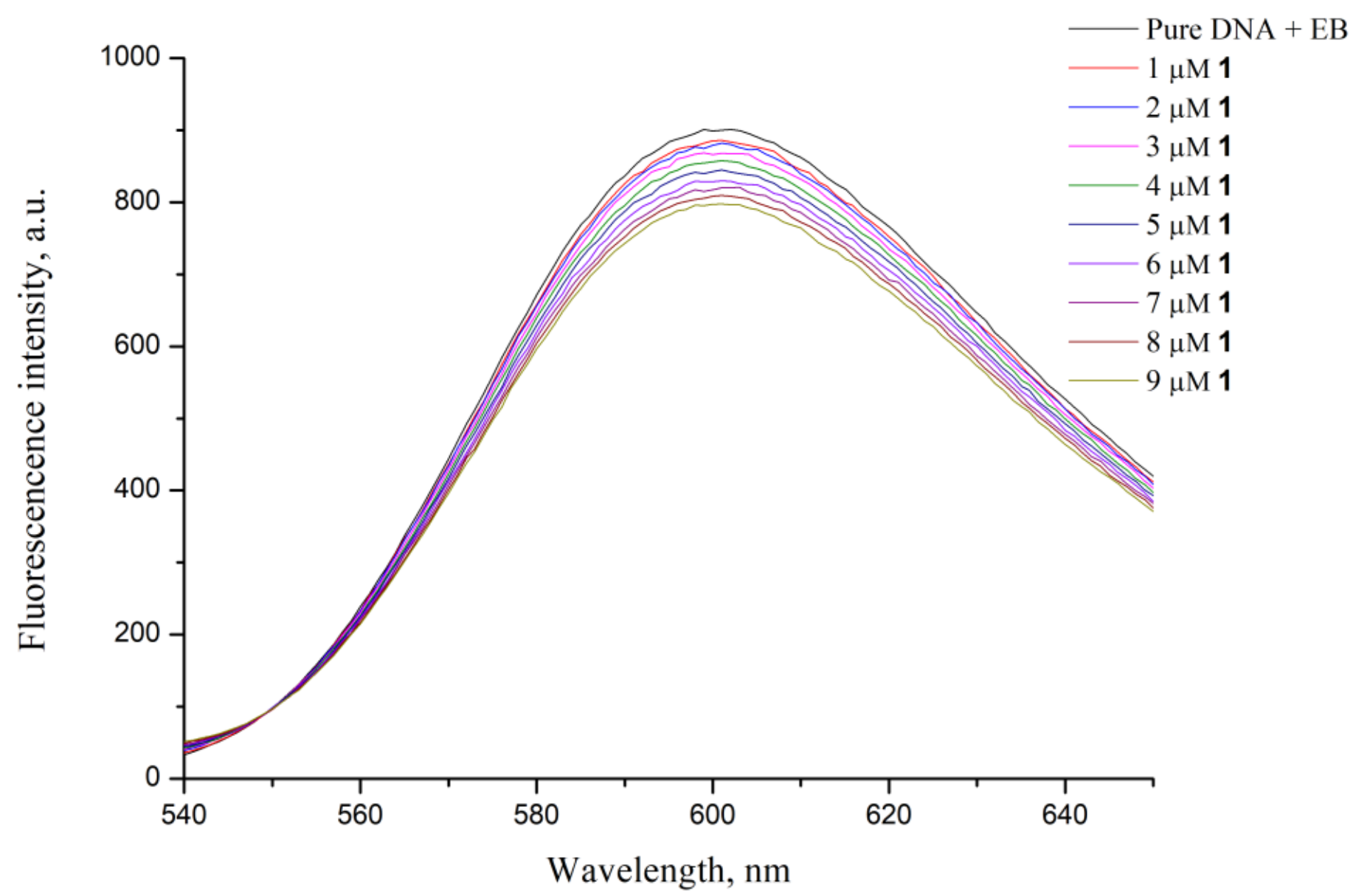

(A)

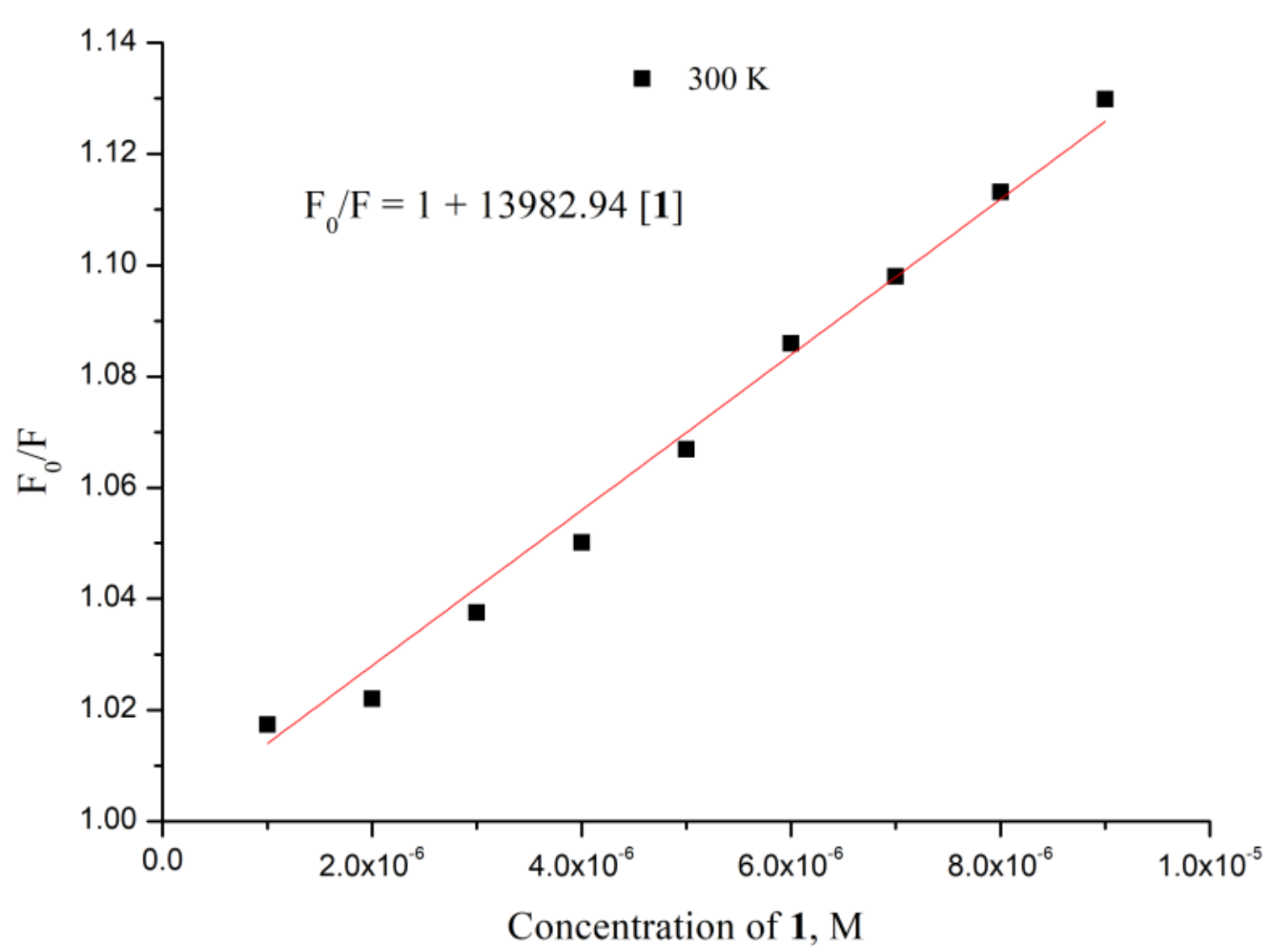

(B)

Supplementary Figure 4. Fluorescence emission spectra of CT-DNA-EB for the titration with the (A) and the Stern–Volmer dependency of intensity on the concentration of **1** (B).

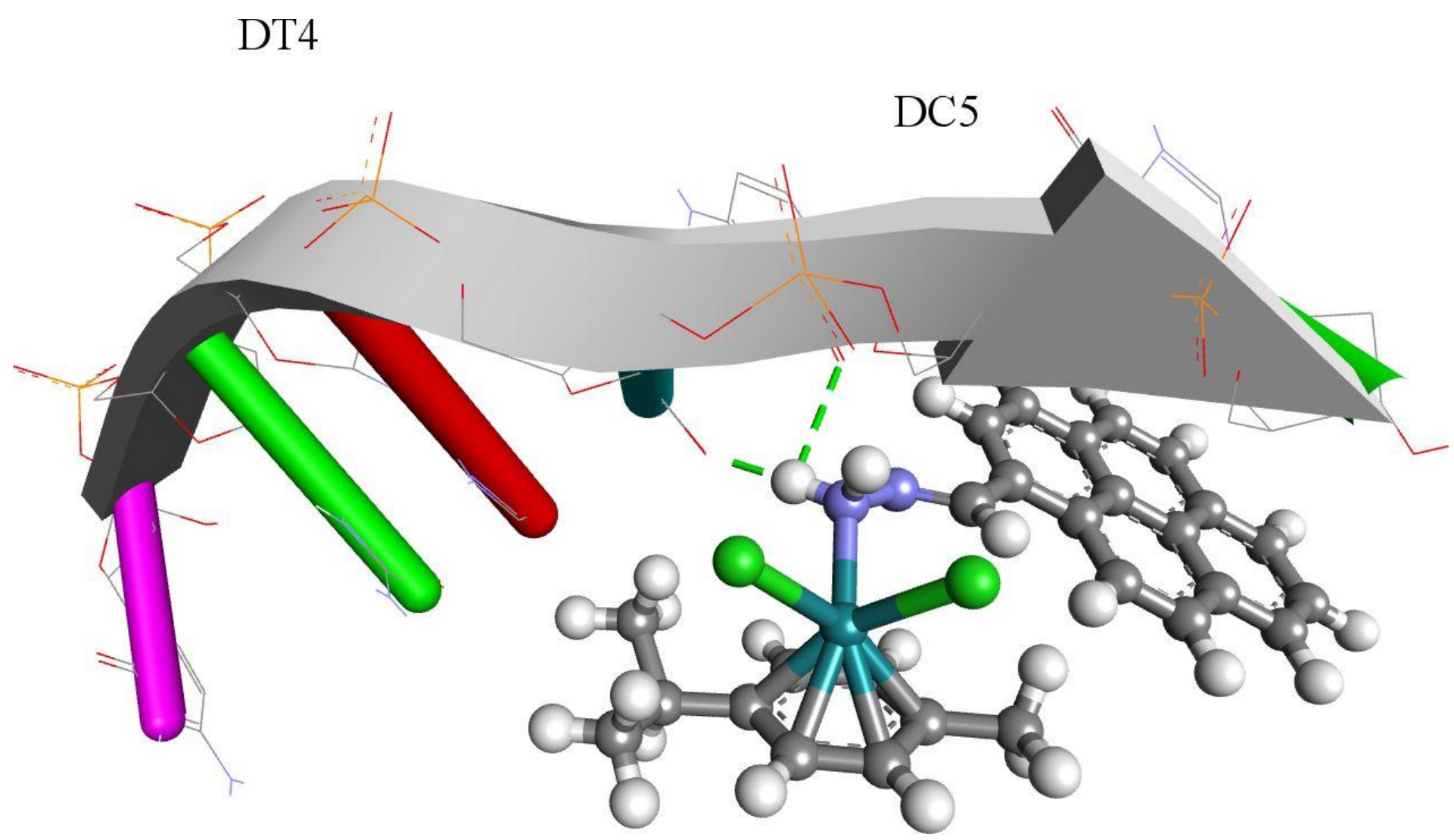

Supplementary Figure 5. Hydrogen bond (green dotted lines) docking interactions of the most stable conformations of selected compound with DNA intercalation.

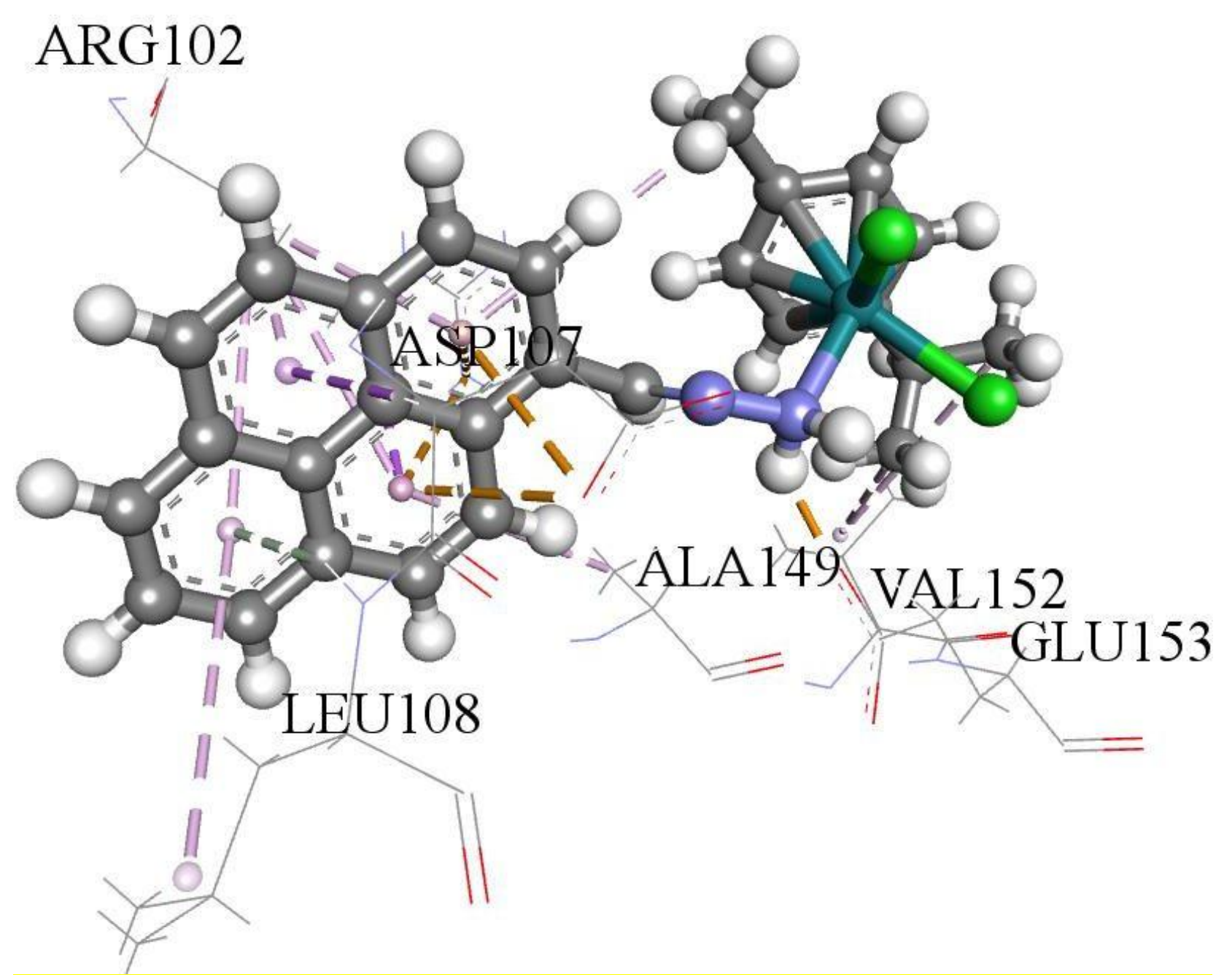

Supplementary Figure 6. Hydrophobic (pink and orange dotted lines) and hydrogen bond (green dotted lines) docking interactions of the most stable conformations of **1** with BCL-2/BCL-xL.

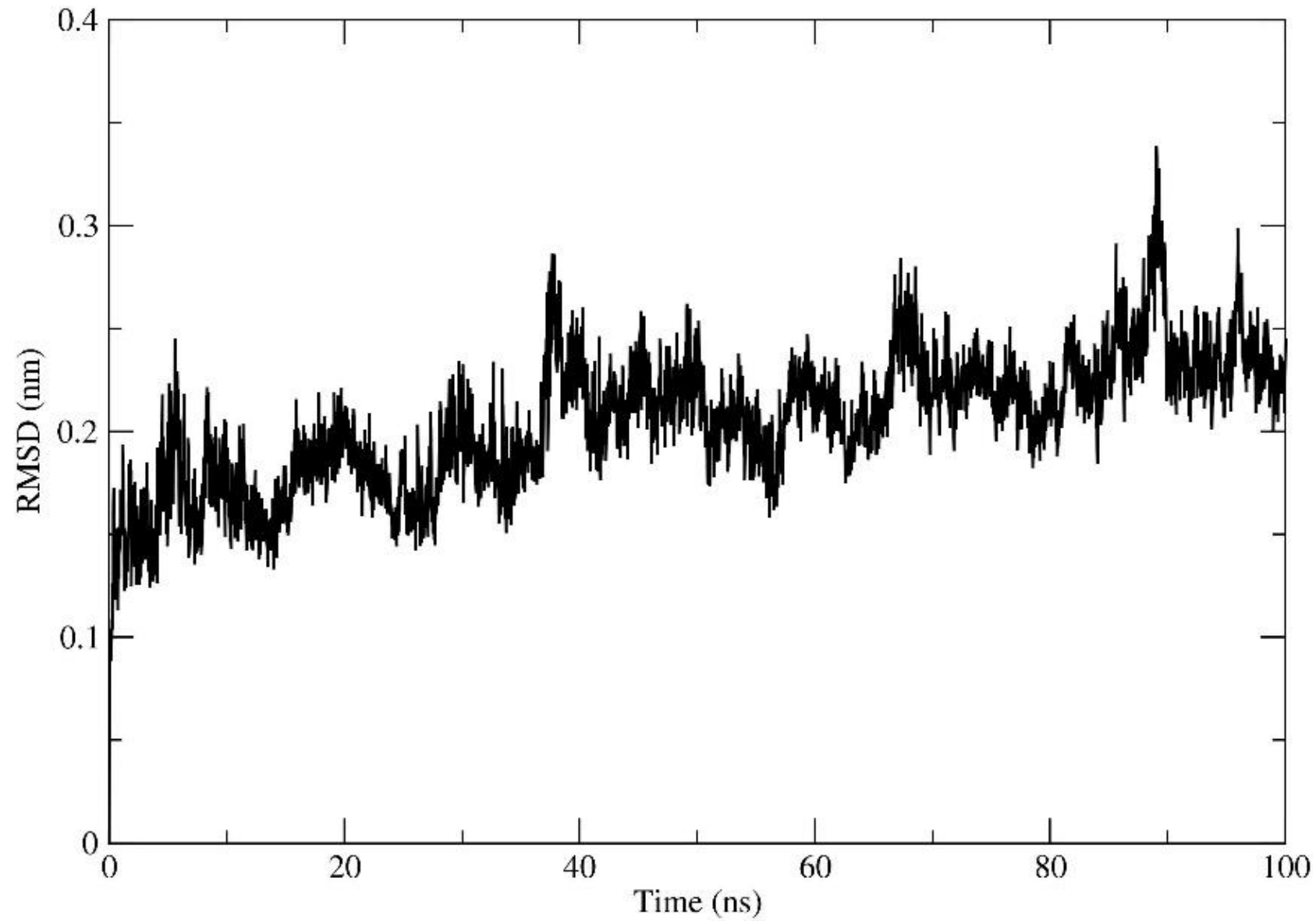

Supplementary Figure 7. Plot of root mean square deviation (RMSD) of C–Cα–N backbone vs. simulation time for solvated BCL-2 in complex with the **1** during 100 ns molecular dynamics simulations.

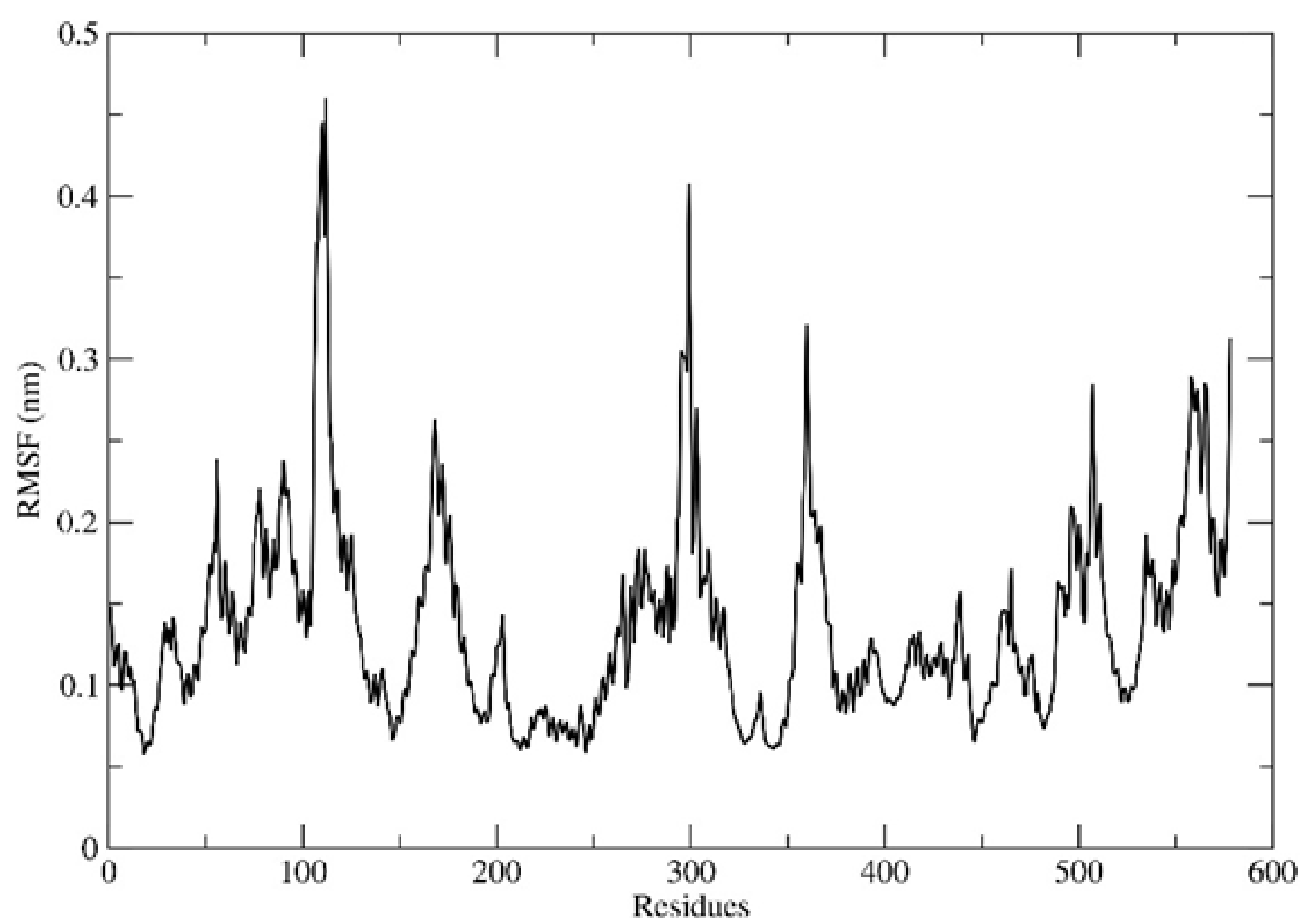

Supplementary Figure 8. The root mean square fluctuation (RMSF) values of BCL-2 in complex with the **1** were plotted against residue numbers.

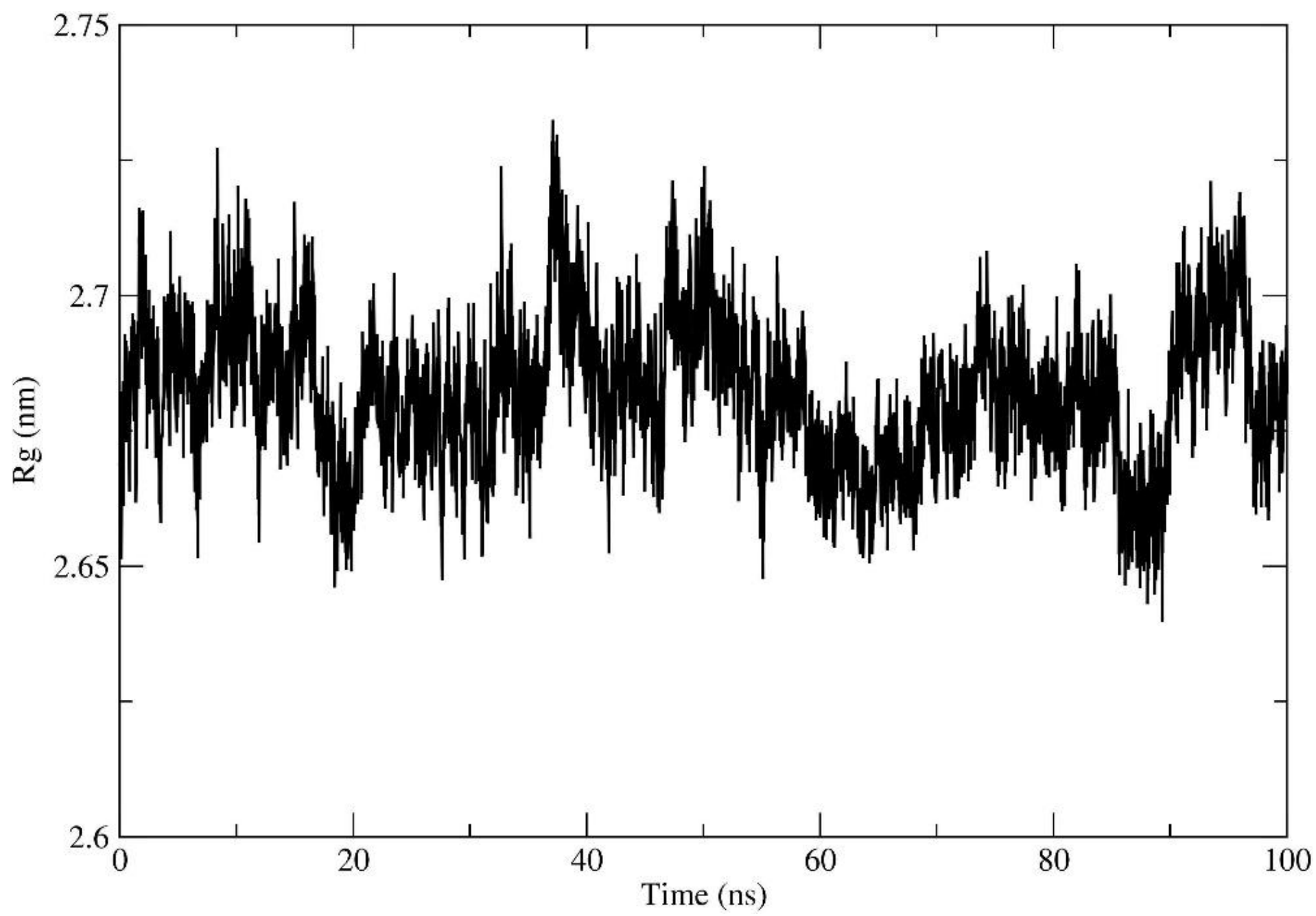

Supplementary Figure 9. Plot of radius of gyration (Rg) during 100 ns MD simulation of BCL-2 in complex with **1**.

Supplementary Table 2. Important thermodynamic parameters during 100 ns MD simulation generated with MMPBSA protocol.

**Complex**	**ΔE_{elec} (kJ/mol)**	**ΔE_{VDW} (kJ/mol)**	**ΔG_{polar} (kJ/mol)**	**$\Delta G_{nonpolar}$ (kJ/mol)**	**$\Delta G_{binding}$ (kJ/mol)**
**1 – BCL-2/BCL-xL**	-331.0±283.3	-106.3±13.2	378.4±282.1	-13.5±1.5	-72.38±14.7

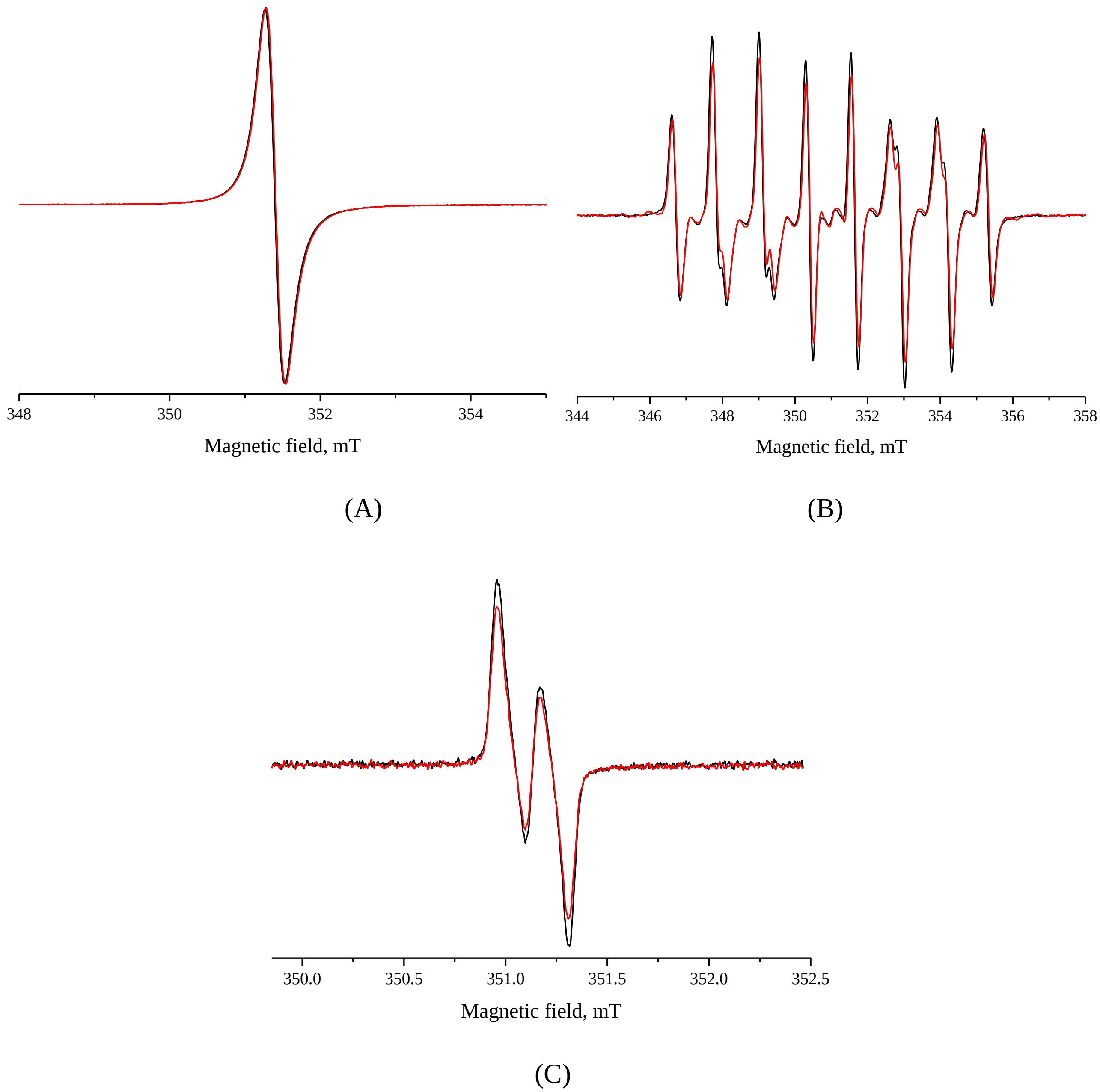

Supplementary Figure 10. The EPR spectra of DPPH (A), DEPMPO/OOH (B), and the ascorbyl radical (C) recorded in the absence (control, black) and presence of **1** (red).

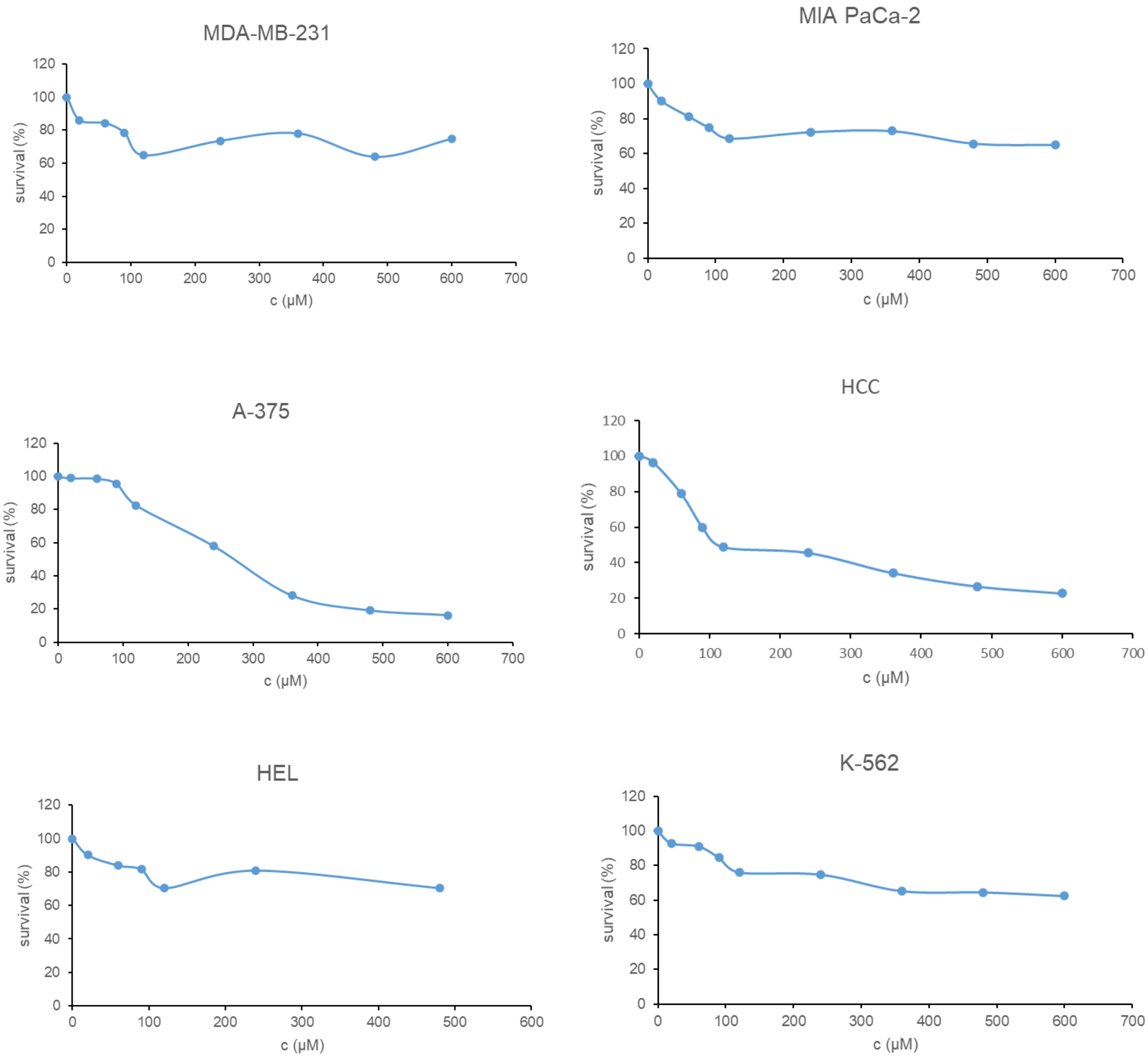

Supplementary Figure 11. Concentration-dependent effects of **1** on the viability of MDA-MB-231, MIA PaCa-2, A-375, HCC, HEL and K-562 cells determined by the MTT assay after 24 h of treatment.

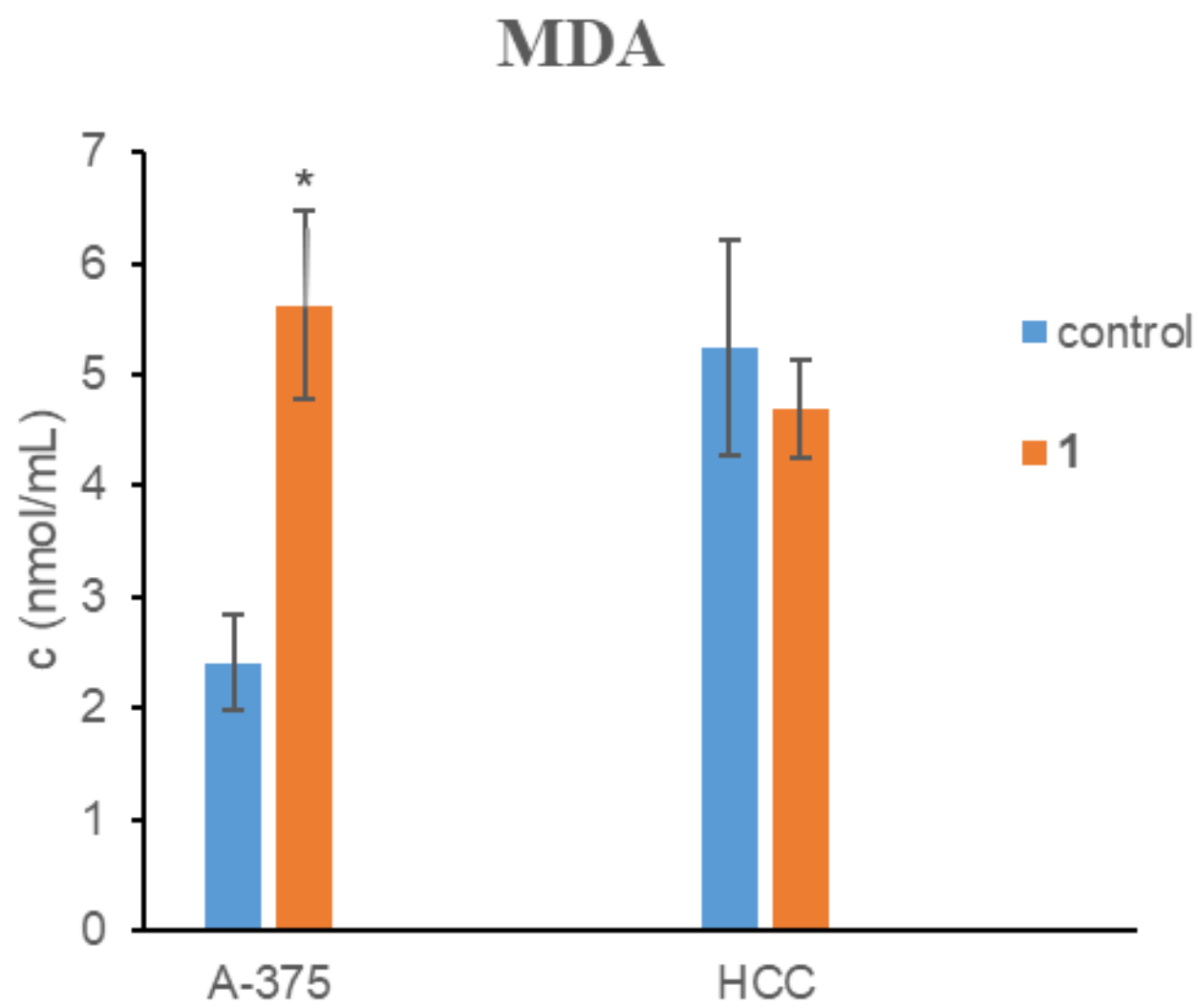

Supplementary Figure 12. Effect of **1** treatment on malondialdehyde (MDA) levels in A-375 and HCC cell lines. Data are presented as mean ± SD. *p < 0.05 compared to control.

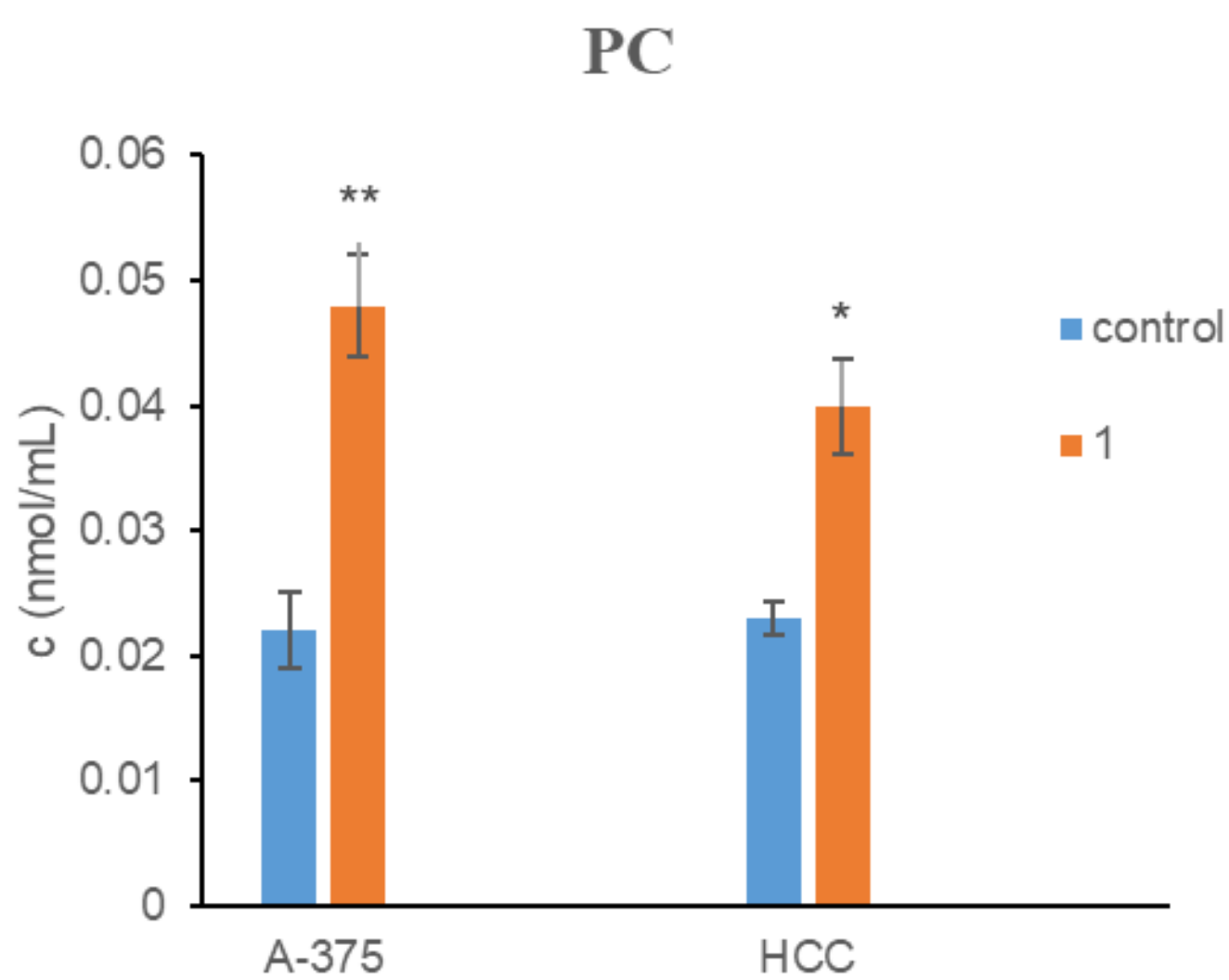

Supplementary Figure 13. Effect of **1** treatment on protein carbonyl (PC) levels in A-375 and HCC cell lines. Data are presented as mean ± SD. *p < 0.05 and **p < 0.01 compared to control.

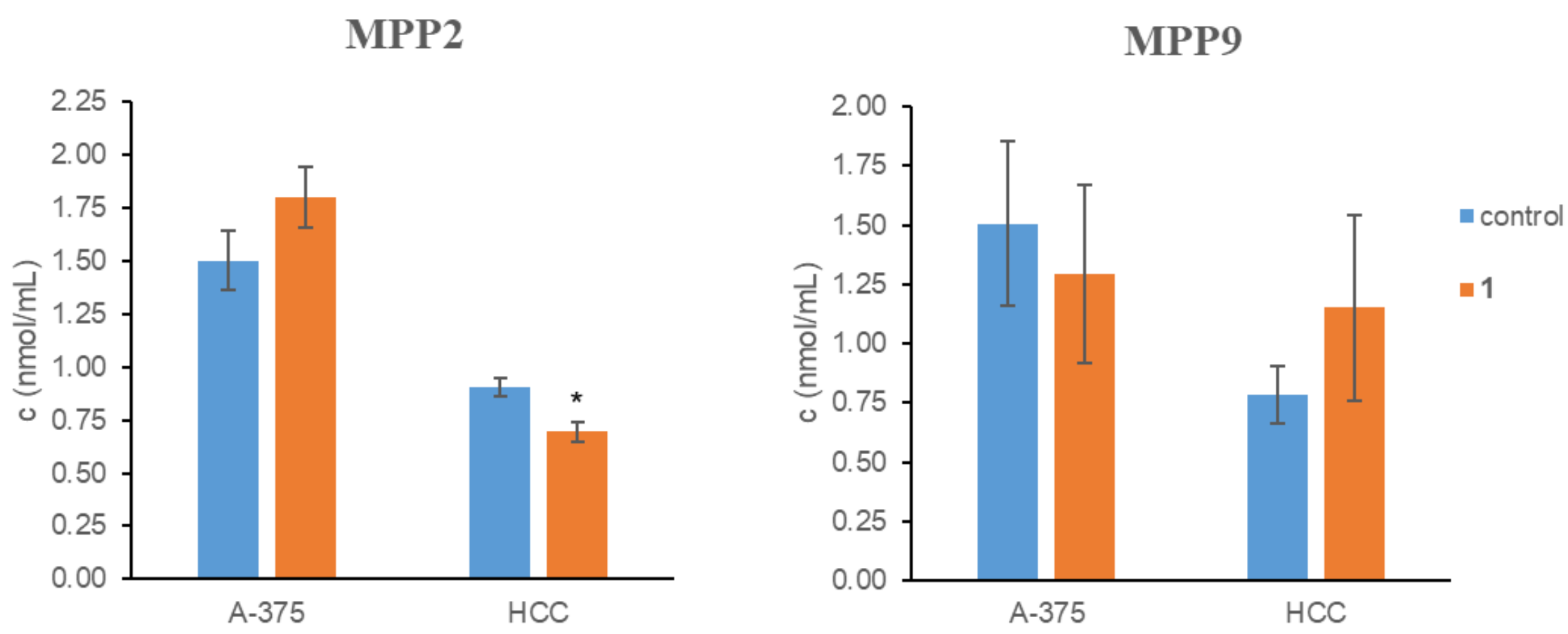

(A) (B)

Supplementary Figure 14. Matrix metalloproteinase 2 (A) and 9 (B) activity in A-375 and HCC cells under the effect of **1** treatment. *p < 0.05 compared to control.
